# Social Support Predicts Differential Use, but not Differential Effectiveness, of Expressive Suppression and Social Sharing in Daily Life

**DOI:** 10.1007/s42761-022-00123-8

**Published:** 2022-08-22

**Authors:** Lisanne S. Pauw, Hayley Medland, Sarah J. Paling, Ella K. Moeck, Katharine H. Greenaway, Elise K. Kalokerinos, Jordan D. X. Hinton, Tom Hollenstein, Peter Koval

**Affiliations:** 1 Department of Psychology, University of Münster, Münster, Germany; 2 Melbourne School of Psychological Sciences, The University of Melbourne, Parkville, Australia; 3 School of Behavioural and Health Sciences, Australian Catholic University, Fitzroy, Australia; 4 Department of Psychology, Queen’s University, Kingston, Canada; 5 Faculty of Psychology and Educational Sciences, KU Leuven, Leuven, Belgium

**Keywords:** Emotion regulation, Context, Wellbeing, Social support, Psychological flexibility

## Abstract

While emotion regulation often happens in the presence of others, little is known about how social context shapes regulatory efforts and outcomes. One key element of the social context is social support. In two experience sampling studies (*N*s = 179 and 123), we examined how the use and affective consequences of two fundamentally social emotion-regulation strategies—social sharing and expressive suppression—vary as a function of perceived social support. Across both studies, we found evidence that social support was associated with variation in people’s use of these strategies, such that when people perceived their environments as being higher (vs. lower) in social support, they engaged in more sharing and less suppression. However, we found only limited and inconsistent support for context-dependent affective outcomes of suppression and sharing: suppression was associated with better affective consequences in the context of higher perceived social support in Study 1, but this effect did not replicate in Study 2. Taken together, these findings suggest that the *use* of social emotion-regulation strategies may depend on contextual variability in social support, whereas their *effectiveness* does not. Future research is needed to better understand the circumstances in which context-dependent use of emotion regulation may have emotional benefits, accounting for personal, situational, and cultural factors.

**Supplementary Information:**

The online version contains supplementary material available at 10.1007/s42761-022-00123-8.

People regulate their emotions in myriad ways, some of which can be done alone, such as when people distract themselves or change their thoughts about an emotional situation. However, other emotion-regulation strategies, such as talking about emotions (*social sharing*) or hiding one’s emotional expressions (*expressive suppression*), are fundamentally social. That is, they involve communicating emotions to—or concealing emotions from—others. Prior research shows that the ability to flexibly share and suppress one’s emotions is crucial for long-term wellbeing (Bonanno et al., 2004; Chen et al., 2018; Westphal et al., 2010). It is theorized that while some emotion-regulation strategies may be overall more or less adaptive, their outcomes are context-dependent and are thus best used in accordance with situational demands (e.g., Aldao et al., 2015; Bonanno & Burton, 2013; Kashdan & Rottenberg, 2010). While influential, these theoretical accounts of flexible emotion regulation have received relatively scant empirical attention, especially in daily life (Colombo et al., 2020; Kobylińska & Kusev, 2019). To address this gap, we investigated how perceived social support, a key socio-contextual factor, predicts the use and short-term affective consequences of sharing and suppression in daily life across two experience sampling studies.

## Sharing and Suppression in Daily Life

Experience sampling and daily diary studies have shown that suppression and sharing are both used commonly in daily life (Cameron & Overall, 2018; Heiy & Cheavens, 2014) and can be best conceptualized as distinct emotion-regulation strategies each with unique intra- and inter-personal correlates (e.g., Cameron & Overall, 2018). However, to our knowledge, no previous studies have explored how these social emotion-regulation strategies vary as a function of changes in perceived social support, a key feature of social contexts, which we define as a person’s subjective evaluation of the degree to which others are available to support them in a given situation (see Eagle et al., 2019) and refer to as ‘social support’ henceforth. Prior research and theory suggests that the social context, and in particular the availability of social support, should influence the degree to which people share or hide their emotions from others (English et al., 2017; Reis, 2008). For example, people tend to share their emotions more with people who are perceived as more responsive and supportive (Clark & Finkel, 2005; Pentina & Zhang, 2017; Ruan et al., 2020; Von Culin et al., 2018). Conversely, people tend to suppress their emotional expressions more in the company of non-close others (English et al., 2017), perhaps because they perceive them as less supportive than close others. Furthermore, prior correlational research shows that emotional suppression is associated with reduced social support and acceptance by others, whereas expression has been associated with enhanced acceptance by others (Cameron & Overall, 2018; Chervonsky & Hunt, 2017). These effects may be partially driven by differential emotion regulation use as a function of the supportiveness of the social environment. Taken together, these findings suggest that greater social support will predict more sharing and less suppression of emotions.

The affective consequences of suppression and sharing may also vary by context. Overall, daily life studies of suppression show that it predicts worse emotional outcomes in the form of greater negative affect (NA) and less positive affect (PA), although the size of these effects varies across studies (Brans, Koval, Verduyn et al., 2013; Brockman et al., 2017; Farmer & Kashdan, 2012; Impett et al., 2012; Nezlek & Kuppens, 2008; but see Heiy & Cheavens, 2014). The short-term affective consequences of social sharing are more mixed (Brans, Koval, Verduyn et al., 2013; Cameron & Overall, 2018; Heiy & Cheavens, 2014). In line with theoretical accounts of regulatory flexibility (e.g., Aldao, 2013; Bonanno & Burton, 2013), these mixed findings may be explained by the affective outcomes of emotion regulation being context-dependent. Specifically, we argue that social support may influence the short-term affective consequences of sharing and suppressing emotions.

We see two contrasting ways that social support could affect the consequences of suppression. One possibility is that the generally harmful affective consequences of suppression may be reduced in contexts of high (compared to low) social support. This would align with research on social support as a buffer for other emotionally harmful psychological states, such as stress (e.g., Cohen & Wills, 1985). To the extent that social support represents a broad coping resource, it may provide benefits for wellbeing that counteract the otherwise harmful effects of suppression. Perceiving others as supportive may increase people’s subjective coping potential, thereby buffering the potentially contra-hedonic emotional consequences of suppression. Another possibility, however, is that hiding emotions from others may be less harmful—or even beneficial—in contexts lacking social support, where the experience of emotion may be met with disapproval and thus is more appropriately inhibited. By reflecting (potentially thwarted) needs, emotions signal vulnerability (Fridlund, 1994; Keltner & Haidt, 1999; Van Kleef, 2009). Consequently, in unsupportive contexts, the expression of emotions may risk being neglected, rejected, or even exploited (Burleson & Goldsmith, 1998; Derks et al., 2008). Under such circumstances, actively suppressing emotions may result in more favorable outcomes. Consistent with this view, suppression appears to be less harmful in cultures that discourage emotional expression (Soto et al., 2011); and at times when people perceive their social status as being low (Catterson et al., 2017)—contexts in which social support may be lacking. Thus, although previous theory and research suggest that social support is likely to influence the affective consequences of suppression, the direction of this moderating effect remains unclear.

A clearer argument can be made about the context-dependent consequences of social sharing: Lab studies have consistently shown that social sharing is perceived as more helpful when the listener acts supportively (Rimé et al., 2020). Furthermore, daily experiences of negative emotion tend to be shorter when they are shared with a supportive partner (Brans, Van Mechelen, Rimé et al., 2013). Thus, we propose that sharing should have greater affective benefits when used in everyday contexts with high (vs. low) social support.

## The Present Research

We sought to test theorizing on regulatory flexibility (e.g., Bonanno & Burton, 2013) by investigating how the use and efficacy of social sharing and suppression vary as a function of social support in daily life. First, we investigated how social support predicts the use of each strategy, hypothesizing that higher levels of social support would be associated with decreased suppression (H1a) and increased sharing (H1b). Second, we investigated the affective consequences of each strategy, predicting that suppression would be associated with decreases in PA and increases in NA (H2a). In contrast, given previous mixed findings, we made no predictions regarding the overall affective consequences of sharing (H2b). Finally, we investigated the moderating effect of social support on the efficacy of each strategy. We made no predictions regarding how social support would moderate the affective consequences of suppression (H3a), but we expected social support to be associated with increased emotional benefits of social sharing (H3b). We tested our hypotheses in an existing experience-sampling dataset (*N* = 179) and sought to replicate our findings in a second, independent experience-sampling dataset (*N* = 123).

## Study 1

### Method

Below we report only methodological details relevant to the current report. For more information, including participant eligibility criteria and data cleaning procedure, see Grommisch et al. (2020). A list of all measured constructs can be found in the Supplemental Materials.

#### Participants

We recruited an adult community sample in Melbourne, Australia (*N* = 186), using online and printed advertisements. After the initial lab session, one participant was deemed ineligible and six others withdrew voluntarily, leaving a final sample of *N* = 179 (65% female) aged 18 to 69 years (*M* = 27.02, *SD* = 8.98).

#### Procedure

Participants attended an introductory lab session during which they completed several questionnaires and downloaded *SEMA2*, a custom-built experience-sampling smartphone application (Harrison et al., 2017).^1^ Over the next 21 days, participants were prompted to complete an experience-sampling survey every 80 (± 30) minutes between 10:00am and 10:00pm, resulting in approximately nine surveys per day. Compliance with the experience-sampling protocol ranged between 35% and 100% (*M* = 84%, *SD* = 13%). At the end of the 21 days, participants were thanked and reimbursed on a pro-rata basis, up to $150 AUD, contingent upon their completion of the experience-sampling protocol and other study components.

#### Measures

Each experience-sampling survey began by asking participants to rate their current feelings on three positive affect items (PA; ‘happy’, ‘confident’, ‘relaxed’) and three negative affect items (NA; ‘sad’, ‘angry’, ‘stressed’), presented in random order at each survey. Momentary PA and NA scores were computed by averaging the positive and negative items, respectively. Multilevel composite reliability (*ω*), estimated with the *multilevelTools* package (Wiley, 2020) following Geldhof et al. (2014), was good for PA (*ω*_within_= .73, *ω*_between_= .93) and NA (*ω*_within_= .62, *ω*_between_= .89).

After rating their affect, participants responded to 10 items, presented in a random order, measuring their use of emotion-regulation strategies^2^ “since the last survey”, which began with the stem “How have you managed your emotions since last survey? Please rate how much you’ve used each strategy to increase, decrease or maintain your level of positive or negative emotions, regardless of whether it worked.” Expressive suppression (“I was careful not to express my emotions to others”) and social sharing (“I talked with someone about my emotions”), were measured with items similar to those used in other experience-sampling studies of emotion-regulation in daily life (e.g., Brans, Koval, Verduyn et al., 2013). Finally, participants were asked to rate several items measuring appraisals of “the situation/environment [they had] been in since last survey,” including an item assessing the extent to which they felt “supported by others in the situation” to measure social support. All experience-sampling items were rated on a scale from 0 (*not at all*) to 100 (*very much*).

#### Data-Analytic Approach

To account for the two-level structure (experience-sampling surveys nested within participants) we analyzed data using multilevel regression using the *lme4* package (Bates et al., 2015) in R (version 4.1.0), with p-values calculated using *lmerTest* (Kuznetsova et al., 2017). Our models included random intercepts and slopes for all Level-1 predictors, which were allowed to correlate freely at Level-2. All Level-1 predictors were within-person standardized to ensure that they represented purely within-person effects and to facilitate model convergence (Wang et al., 2019). However, when specific models did not converge using the lme4 default settings, we re-ran these models using the “bobyqa” optimizer allowing for a maximum of 200,000 iterations (Bates et al., 2015). All models included the lagged outcome (excluding overnight lags) as a Level-1 covariate to model change in the outcome as a function of the key predictors over and above persistence in the outcome across successive measurement occasions. We followed-up statistically significant interactions by plotting simple slopes at ± 1 *SD* around the mean level of the moderator. We note that our data are correlational and thus do not allow us to make strong inferences about the causal direction of our findings. Where relevant, we ran supplemental analyses to test whether effects may flow in the opposite direction and we acknowledge potential threats to our causal inferences in the Discussion.

### Results

Descriptive statistics and within- and between-person correlations are shown in Table 1. Key model estimates related to our hypotheses are shown in Table 2. Estimates of all fixed effects for the main analyses can be found in Tables S1-S5 in the online supplemental materials.

**Table 1:** Descriptive statistics study 1: between-person means (*M*), within-person (*SDw*) and between-person standard deviations (*SDb*), intraclass correlation coefficients (ICC) and between-person and within-person correlations

Variable	*M*	*SDw*	*SDb*	ICC	Sharing	Suppression	Support	NA	PA
Sharing	35.37	25.84	19.82	.35	–	.50***	.57***	.22**	.34***
Suppression	38.54	22.73	22.75	.46	-.07***	–	.20**	.43***	.04
Social Support	56.36	23.98	16.20	.29	.34***	-.08***	–	-.16*	.58***
Negative Affect (NA)	22.10	13.33	13.38	.47	.00	.12***	-.19***	–	-.46***
Positive Affect (PA)	62.88	14.77	13.32	.42	.10***	-.09***	.27***	-.63***	–

*Note.* * *p* < .05; ** *p* < .01; *** *p* < .001. Between-person correlations are shown above the diagonal; average within-person correlations are below the diagonal

**Table 2:** Fixed effect estimates for hypothesized effects in Study 1

Hypothesis	Outcome	Predictor	Estimate (SE)	95% CI	*p*
H1a	Expressive Suppression	Social Support	-1.48 (0.42)	[-2.30, -0.65]	**.001**
H1b	Social Sharing	Social Support	8.27 (0.45)	[7.38, 9.16]	**< .001**
H2a	Positive Affect (PA)	Suppression	-0.81 (0.19)	[-1.19, -0.43]	**< .001**
	Negative Affect (NA)	Suppression	1.28 (0.17)	[0.96, 1.61]	**< .001**
H2b	Positive Affect (PA)	Sharing	1.26 (0.18)	[0.92, 1.61]	**< .001**
	Negative Affect (NA)	Sharing	-0.05 (0.16)	[-0.37, 0.26]	.733
H3a	Positive Affect (PA)	Suppression * Social Support	0.17 (0.11)	[-0.06 – 0.39]	.142
	Negative Affect (NA)	Suppression * Social Support	-0.24 (0.11)	[-0.45 – -0.02]	**.032**
H3b	Positive Affect (PA)	Sharing * Social Support	0.22 (0.13)	[-0.04 – 0.47]	.100
	Negative Affect (NA)	Sharing * Social Support	-0.19 (0.12)	[-0.43 – 0.05]	.132

First, we examined whether momentary use of suppression and social sharing varied as a function of social support. Supporting Hypotheses 1a and 1b, we found that greater social support predicted decreased use of suppression, and increased use of social sharing.

Next, we examined the affective consequences of the two regulatory strategies. Supporting Hypothesis 2a, momentary suppression predicted higher NA and lower PA. Social sharing, on the other hand, predicted an increase in PA, but had no effect on NA.

**Fig. 1:**
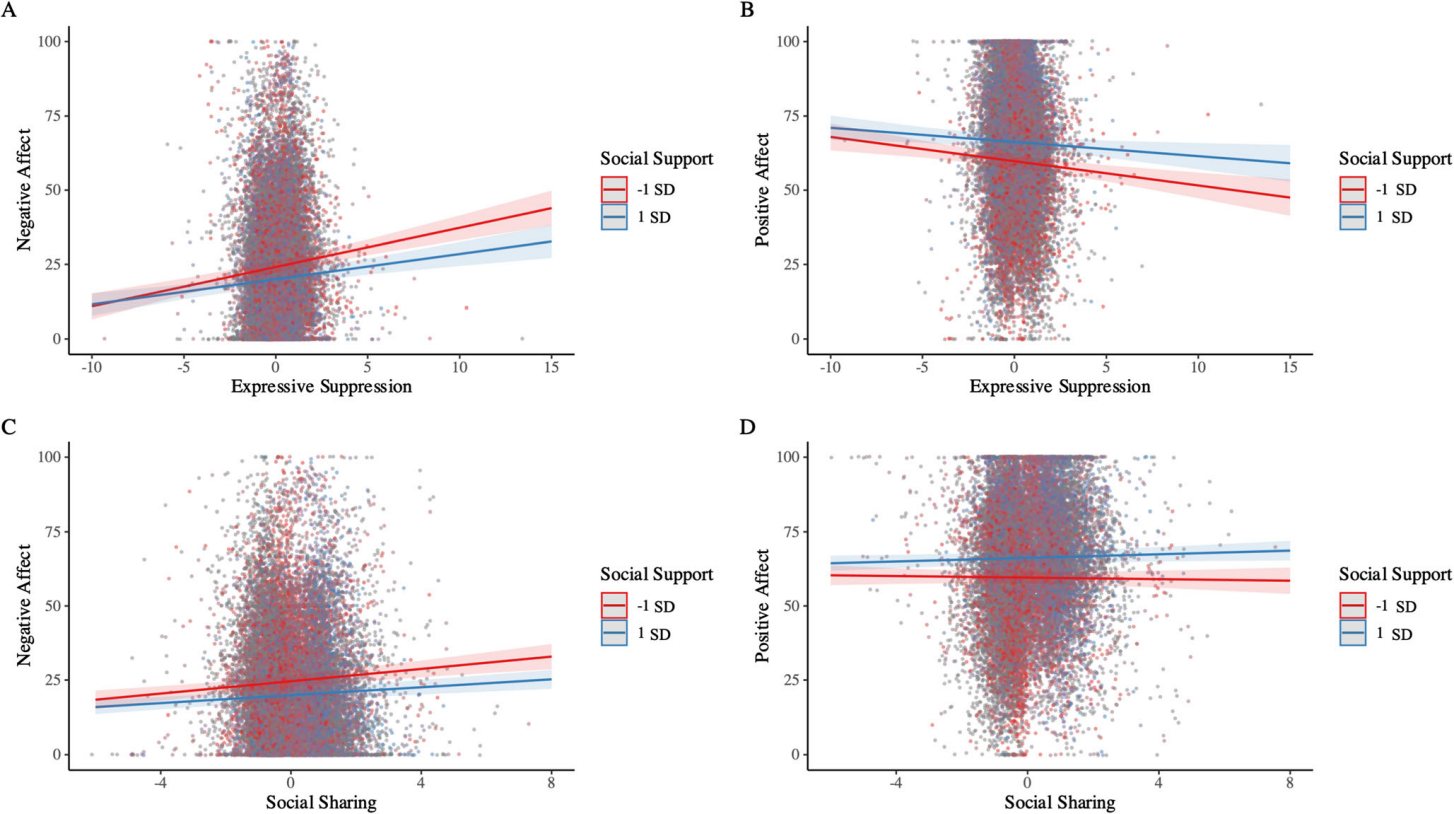
Simple slope plots illustrating the moderating effect of social support on the short-term affective consequences of expressive suppression and social sharing on negative affect and positive affect in Study 1. Simple slopes are plotted at ± 1 *SD* around the mean level of social support. Panel A (interaction between social support and suppression predicting negative affect) was statistically significant at *p* < .05. No other interactions were statistically significant

Finally, we examined whether the affective consequences of suppression and sharing were moderated by perceived levels of support. Regarding Hypothesis 3a, social support did not moderate the effect of suppression on PA. However, we found a small, yet statistically significant interaction among social support and suppression for NA. As displayed in Fig. 1 (Panel A), suppression predicted smaller increases in NA in contexts with higher (vs. lower) levels of social support. Thus, social support appeared to buffer the harmful consequences of suppression on negative affect. Contrary to Hypothesis 3b, there was no significant interaction between social sharing and social support predicting NA or PA.

#### Supplemental Analyses

To test the robustness of the key findings from our main analyses, we ran a series of additional analyses reported in the online supplemental materials. First, we examined the specificity of the effects for social sharing and suppression. To establish whether the effects we observed for each strategy (i.e., suppression or sharing) were independent of the other strategy, we repeated our main analyses controlling for the effect of the other regulation strategy in each model. That is, in each model including suppression as an outcome (H1) or a focal predictor (H2 and H3), we added social sharing as a Level-1 covariate, whereas in models including sharing as an outcome (H1) or a focal predictor (H2 and H3) we controlled for the effect of suppression at Level-1 (see Tables S6-S8). Our key findings were robust in these models, except for the interaction between social support and suppression predicting NA, which was no longer statistically significant when controlling for sharing (see Table S8 in Supplement 2.1).

Furthermore, we ran additional analyses examining whether social support predicted differential use of three other ‘non-social’ emotion-regulation strategies (i.e., reappraisal, rumination, distraction) to examine the specificity of our main findings to social—rather than less social—emotion-regulation strategies. These analyses showed that social support was associated with greater use of reappraisal and distraction, but not with rumination (see Table S9). While distraction and reappraisal are not necessarily social emotion-regulation strategies, it is plausible that the increased use of these strategies was partially the result of social support obtained in response to sharing the emotion (i.e., the other may have distracted the sharer or helped them reappraise the situation).

Second, we ran an additional set of analyses to test the temporal nature and directionality of our findings regarding the influence of social support on suppression and sharing. Specifically, we reran our main analyses for H1 without including lagged suppression or sharing use as covariates. These analyses yield identical findings compared to our main analyses (see Supplement 2.2 and Table S10). Furthermore, given the correlational nature of our main analyses, they do not speak to the direction of the effect. Therefore, we repeated our analyses testing H1 using lagged social support as a predictor. We also investigated the possibility that the effect may flow in the opposite direction by modeling how lagged suppression and sharing predict future social support. These analyses support bidirectional lagged relationships among social support and sharing, but no lagged associations between social support and suppression (see Tables S11 and S12 in Supplement 2.2).

Third, to improve comparability between studies, we re-ran our main analyses of Study 1 with valence as the outcome (Valence = PA – NA). These results mirror those obtained when analysing PA and NA separately (see Tables S13-S14).

Finally, we largely replicated all our main analyses excluding the cases in which participants reported having (mostly) been alone, and explored the potential moderating role of ethnicity (see Tables S15-S20, Supplement 2.3 and 2.4).

## Study 2

To replicate our Study 1 findings, we ran similar analyses using another experience-sampling dataset. This dataset, previously published by Medland et al. (2020), was collected with similar methods to Study 1, except (i) a shorter experience-sampling protocol of 7 days; (ii) suppression and social sharing were measured with two items each, and reported in response to the most intense negative emotional experience in the past hour; (iii) social support was measured using a categorical and a continuous item, which we combined into an overall social support score; and (iv) momentary affect was measured on a single bipolar scale, rather than positive and negative affect scales.

### Method

Below we report only methodological details relevant to the current report. For more information, including participant eligibility criteria, data cleaning procedure and an overview all of measured constructs, see Medland et al. (2020).

#### Participants

We recruited 132 participants via online advertisements, and through an undergraduate research pool at a university in Melbourne, Australia. Four participants voluntarily withdrew and five were excluded due to technical problems, leaving a final sample of *N* = 123 (66.7% female) aged 18 to 34 years (*M* = 21.2, *SD* = 3.5). Participants were reimbursed pro-rata, depending on their experience-sampling compliance. Undergraduate participants received course credit and community participants were reimbursed up to $45 AUD.

#### Procedure

Similar to Study 1, participants attended an introductory lab session during which they completed several questionnaires and downloaded the SEMA2 app onto their smartphones. Over the next 7 days, participants were prompted to complete an experience-sampling survey every 90 (± 20) minutes between 9:00 am and 9:00 pm (i.e., 8-9 surveys per day). Compliance ranged between 7.9% and 100% (*M* = 79.6%, *SD* = 17.5%).

#### Measures

Each experience-sampling survey began by asking participants to rate their current affect (“how are you feeling right now?”) using a bipolar *valence* scale from –10 (*very negative*) to +10 (*very positive*). Next, after participants selected their “strongest negative emotion…in the last hour”, they rated “how intense” this emotion was (*negative emotion intensity*) on a slider scale ranging from 0 (*not at all, I barely noticed*) to 100 (*very intense*). All remaining experience-sampling questions were framed in relation to this recent negative emotional experience.

Social support was measured with two items. First, participants responded to a categorical *support* item (“Did you feel supported by the other person(s) present?”) with response options of “yes,” “no,” or “I was alone.” Next, if participants answered “yes” to the categorical support item (*n* = 1,725), they completed a continuous measure of *support intensity* (“To what extent did you feel supported by the other person(s) present?”) on 0 (*not at all*) to 100 (*very much*) scale. For comparability with Study 1, which included a continuous measure of social support presented at all experience-sampling surveys without asking whether people were alone, we used the continuous support intensity item for our main analyses. However, we replaced missing values for support intensity with 0s when participants responded “no” (*n* = 1,394) or “I was alone” (*n* = 2,843) to the categorical support item. We acknowledge that being alone is not the same as having an unsupportive interaction. Therefore, we repeated our analyses excluding “I was alone” responses in the supplemental materials (see Tables S36 and S37). These analyses replicate our main findings.

Next, participants responded to 12 items (presented in random order) measuring their use of emotion-regulation strategies, which began with the stem “In response to my emotion,” referring to their most intense negative emotion in the past hour. These items were adapted from the Regulating Emotion Systems Survey (RESS; De France & Hollenstein, 2017) to suit experience-sampling (Medland et al., 2020). Social sharing, also referred to as *engagement* (De France & Hollenstein, 2017), was measured with the items “I showed my feelings” and “I expressed my feelings,” whereas suppression was measured with the items “I made an effort to hide my feelings” and “I pretended I wasn’t upset,” all rated on a scale from 0 (*not at all*) to 100 (*very much*). We calculated momentary sharing and suppression composites by averaging scores on the two items assessing each strategy at each experience-sampling survey. Multilevel correlations, estimated using the *psych* package (Revelle, 2021), were adequate for both social sharing (*r*_within_= .64, *r*_between_= .97) and suppression (*r*_within_= .54, *r*_between_= .96). Finally, participants rated their perceived emotion regulation success with the following question: “Overall, how successful were you in regulating your emotions?” on a scale from 0 (*not at all*) to 100 (*very much*).

#### Data-Analytic Approach

Study 2 did not include PA and NA measures equivalent to those in Study 1. Therefore, the data analytic approach was identical to Study 1, except we tested Hypotheses 2 and 3 using bipolar valence as an outcome.

### Results

Descriptive statistics as well as within- and between-person correlations are shown in Table 3. Model estimates for all hypothesized effects are shown in Table 4. Estimates of all fixed effects can be found in Table S21-23 in the Supplemental Materials available online.

**Table 3:** Descriptive statistics Study 2: between-person means (*M*), within-person (*SDw*) and between-person standard deviations (*SDb*), intraclass correlation coefficients (ICC) and between-person and within-person correlations

Variable	*M*	*SDw*	*SDb*	ICC	Valence	Sharing	Suppression	Support
Valence	2.95	3.58	2.94	.36	-	.12	-.20*	.09
Sharing	41.51	20.91	18.05	.38	-.02	-	.24**	.25**
Suppression	37.42	20.15	18.74	.43	-.02	-.15***	-	.05
Social Support	19.86	27.18	16.98	.24	.17***	.19***	-.05***	-

*Note.* * *p* < .05; ** *p* < .01; *** *p* < .001. Between-person correlations are shown above the diagonal; average within-person correlations are below the diagonal. Valence was measured on a scale from -10 (very negative) to +10 (very positive)

**Table 4:** Fixed effect estimates for hypothesized effects in Study 2

Hypothesis	Outcome	Predictor	Estimate (SE)	95% CI	*p*
H1a	Suppression	Social Support	-1.13 (0.47)	-2.05 – -0.21	**.017**
H1b	Sharing	Social Support	3.97 (0.50)	3.00 – 4.94	**<.001**
H2a	Valence	Suppression	-0.16 (0.09)	-0.34 – 0.02	.086
H2b	Valence	Sharing	-0.01 (0.10)	-0.19 – 0.18	.941
H3a	Valence	Suppression * Social Support	-0.01 (0.06)	-0.13 – 0.11	.905
H3b	Valence	Sharing * Social Support	0.03 (0.07)	-0.11 – 0.16	.683

**Fig. 2:**
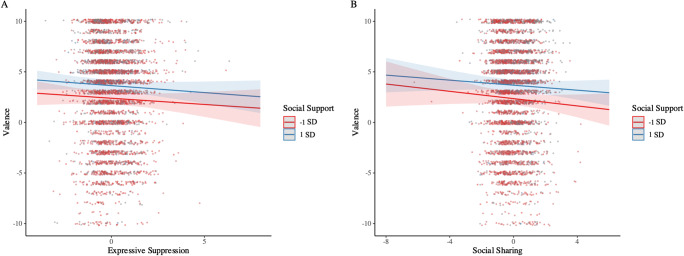
Simple slope plots illustrating the moderating effect of social support on the short-term affective consequences of expressive suppression and social sharing on valence in Study 2. Simple slopes are plotted at ± 1 *SD* around the mean level of social support. None of the interactions were statistically significant at *p* < .05

Supporting Hypotheses 1a and 1b, and replicating our findings in Study 1, higher levels of social support predicted decreased use of suppression and increased use of social sharing. Contrary to Hypothesis 2a and our findings in Study 1, suppression and social sharing were not associated with changes in momentary valence. Finally, contrary to Hypotheses 3a and 3b, and partly inconsistent with Study 1 findings, social support did not moderate the affective consequences of suppression or social sharing (see Fig. 2).

#### Supplemental Analyses

To test the robustness of the key findings from our main analyses, we ran a series of additional analyses, the results of which we report in the supplemental online materials. First, we examined the specificity of our findings to social sharing and suppression. To establish whether the effects we observed for suppression and sharing were independent of the other strategy, we again repeated our main analyses controlling for the effect of the other regulation strategy in each model (see Tables S24-S26). These analyses largely replicate our key findings (see Supplement 2.1 for a more elaborate description of the findings). Furthermore, we ran additional analyses examining whether social support predicted differential use of three other ‘non-social’ emotion-regulation strategies (i.e., reappraisal, rumination, distraction; see Table S27). These analyses showed that social support was associated with greater use of reappraisal, but not with distraction and rumination.

Second, to test the temporal nature and causal directionality of our findings regarding the influence of social support on suppression and sharing, we ran an additional set of analyses. See Study 1 and Supplement 2.2 for a more elaborate description of how and why we performed these analyses. In short, we re-ran our analyses testing H1 *without* lagged ER strategies as covariates. These analyses replicated our main findings (see Table S28). Furthermore, we repeated our analyses testing H1 using lagged social support as a predictor of the two regulatory strategies, and vice versa. Overall, we found no significant lagged effects, suggesting that the effect of social support on the use of suppression and sharing unfolds on a relatively short timescale (see Tables S29 and S30 for the parameter estimates).

Third, we repeated our main analyses with two alternative dependent measures (see Supplement 2.3). Whereas Study 1 included measures of both PA and NA, our analyses of Study 2 were limited to valence. Therefore, we re-ran our analyses testing Hypothesis 2 and 3 using participants’ intensity ratings of their most negative emotional experience in the last hour as an outcome, as an additional test of the (context-dependent) affective consequences of both regulatory strategies (see Tables S31 and S32). These analyses replicated our main analyses, except that both suppression and sharing were associated with an increase in negative emotional intensity. Note, however, that negative intensity was measured in response to the emotional experience that was regulated, suggesting that higher negative emotional intensity may have been the antecedent, rather than consequence, of greater suppression and sharing.

Furthermore, we explored whether context-dependent emotional benefits may be reflected in *perceived* efficacy of emotion-regulation strategies, rather than in the actual affective consequences. To this end, we re-ran our analyses testing Hypothesis 3, replacing affect with perceived emotion regulation success as a dependent measure. These analyses replicated our main findings, except for a significant interaction indicating that participants experienced expressive suppression as more effective in the context of lower social support (see Table S35).

Moreover, given that people may be more likely to seek (and receive) social support when they experience more intense negative emotions, we reran our analyses of Study 2, controlling for negative emotional intensity of the stressor. These analyses replicate our key findings, except that social support was no longer significantly negatively associated with suppression (see Tables S33-S34 and Supplement 2.3 for a more elaborate description of the findings).

Finally, we replicated all our main analyses with an alternative operationalization of social support, in which social support was coded as missing when participants reported being alone on the categorical social support item (see Tables S36 and S37 and Supplement 2.3). Furthermore, we explored the potential moderating role of ethnicity (see Tables S38-S39, Supplement 4).

## Discussion

We sought to test theoretical accounts of flexible emotion regulation (Aldao et al., 2015; Bonanno & Burton, 2013; Sheppes, 2020) by investigating the context-dependent use and efficacy of social sharing and suppression in daily life. As sharing and suppression are fundamentally social emotion-regulation strategies, we reasoned the use and affective consequences of these strategies should depend on a salient feature of the social context, namely social support. Across two experience-sampling studies, we found that use of sharing and suppression indeed vary by social context: when people perceived their environments as being higher (vs. lower) in social support, they engaged in more sharing and less suppression.

The finding that social support was associated with increased sharing and decreased suppression resonates with a wealth of literature on the importance of perceived partner responsiveness. Perceived responsiveness refers to the confidence that the other will understand, accept, and be responsive to one’s needs, and lies at the core of emotional and relational wellbeing (for overviews, see Reis et al., 2017; Reis & Gable, 2015). Prior work shows that social sharing brings about feelings of relief and strengthens interpersonal relationships when listeners respond supportively (Brans, Van Mechelen, Rimé et al., 2013; Graham et al., 2008). Our findings suggest that by selectively sharing or hiding emotions in line with perceived social resources, people may be strategically seeking to increase their chances of obtaining positive outcomes. However, as we discuss below, our findings in relation to the context-dependent affective outcomes of sharing and suppression do not bear this out.

Overall, the associations of sharing and suppression with momentary affect were partially in line with our hypotheses. In Study 1, social sharing showed some benefits (increased PA, no change in NA) and suppression was associated with emotional costs (reduced PA, increased NA). Yet, in Study 2, neither strategy was associated with short-term changes in affect. Our findings for sharing are consistent with past research that has found an overall mixed or null effect of sharing on emotional outcomes (Brans, Van Mechelen, Rimé et al., 2013; Heiy & Cheavens, 2014). Our findings for suppression are consistent with previous daily life studies (e.g., Brans, Koval, Verduyn et al., 2013), but contrast with experimental work suggesting suppression does not influence experience (Kalokerinos et al., 2015).

Contrary to predictions, we found no consistent evidence that social support moderated the affective outcomes of either suppression or sharing. A significant interaction between social support and sharing predicting NA, observed in Study 1, suggested that social support may buffer the harmful consequences of suppression (cf. Cohen & Wills, 1985). However, this effect did not replicate in Study 2 and we found no corresponding moderation for the impact of suppression on PA in either study. Furthermore, our supplemental Study 2 analyses suggested the opposite pattern when predicting *perceived* regulation success, such that expressive suppression was perceived as more effective in the context of lower social support. Thus, overall, our findings do not support the context-dependent affective consequences of either strategy.

Our difficulty in detecting robust context-dependent affective consequences speaks to several challenges faced by the field. So far, most work on emotion regulation flexibility has been theoretical (Kobylińska & Kusev, 2019), featuring large heterogeneity in conceptualizations and operationalizations of flexibility (Aldao et al., 2015). While research examining individual differences in flexibility speaks to a wide variety of psychological health benefits (Bonanno & Burton, 2013; Kashdan & Rottenberg, 2010), a clear mapping of which strategies are best for particular people in particular situations is still lacking (Doré et al., 2016). Absent any overall effects, we speculate that for some people, suppression is more effective when support is high, whereas for others it may be more effective when support is low, due to individual or cultural differences (see e.g., Le & Impett, 2013). For example, individuals who are more sensitive to social signals (e.g., those high in rejection sensitivity, high in social anxiety, or low in self-esteem) may be better off suppressing their emotions in contexts where emotions would be met with low social support. In line with this notion, prior work shows that the relational costs and benefits of sharing emotions with romantic partners are influenced by one’s social anxieties and desire to avoid rejection (Kashdan et al., 2007).

Similarly, the potential moderating role of social support in shaping the affective consequences of social sharing may depend on other situational factors that were not measured in the present studies, such as the specific type of support provided. For example, it is plausible that participants primarily reported high levels of perceived support when obtaining a high degree of emotional support (see Pauw et al., 2018): Prior research shows that emotional support alone is not enough to improve affect regarding the shared emotional experience. Instead (or in addition), attempts to help the sharer to reappraise the situation (i.e., cognitive support) may be necessary for social sharing to be effective (Batenburg & Das, 2014; Nils & Rimé, 2012).

A challenge for future research will thus be to examine these various factors across multiple studies to contribute to a more sophisticated and comprehensive understanding of emotion-regulation flexibility. Importantly, understanding when strategies are not helpful (or not harmful) constitutes an important part of this puzzle. Null findings should therefore not be neglected (Aldao et al., 2015).

### Limitations and Future Directions

We note several limitations of the current studies. First, our studies relied on self-report. While experience-sampling reduces recall biases, it may still be difficult for participants to accurately report on contextual factors, such as social support, or even their own emotion regulation efforts, which may sometimes be implicit or automatic (Braunstein et al., 2017; Gyurak et al., 2011).

Second, in Study 1, emotion regulation and social support were assessed retrospectively (i.e., since the last survey). As such, we cannot be certain that ratings of social support referred to the same context as the emotion-regulation episode. This limitation was mitigated, to an extent, in Study 2 where participants reported their use of regulation strategies and social support in relation to the same (most intense) recent negative emotional episode. Given this focus on the most intense negative experience, it is possible that Study 2 tracked more ineffective regulation, which might explain why we did not replicate the associations of suppression and sharing with momentary affect observed in Study 1. Nevertheless, it should be noted that the mean for negative emotional intensity was below the scale midpoint, suggesting that participants were typically reporting on moderately unpleasant emotions that should be representative of daily life experiences. Furthermore, analyses controlling for emotional intensity replicated our main findings (see Tables S33-S34). Thus, while each study is limited in different ways, they examine instances of varying regulatory need and therefore increase the generalizability of our findings.

Third, our constructs were measured somewhat differently across both studies. For example, social sharing was operationalized as *talking* about negative emotions in Study 1, and as *expressing* negative emotions in Study 2. There are some clear commonalities across these measures that allowed us to examine the robustness of our findings: We replicated the association between greater social sharing in contexts of high (vs. low) social support across both operationalizations of social sharing. However, there are also some important differences across these measures, which may partially explain why social sharing was associated with enhanced positive affect in Study 1, but not in Study 2: Mere expression of emotions may not suffice to elicit positive affect, whereas talking about one’s emotions may.

Furthermore, whereas participants in Study 2 were instructed to report on an instance of regulating *negative* emotions, Study 1 did not include such specific instructions and may thus represent the regulation of both positive and negative emotions. However, given that prior research shows that people much more often hold goals to regulate negative (rather than positive) emotions in daily life (Riediger et al., 2009), we believe our findings mostly reflect the regulation of negative emotions. Future research is needed to examine potential differences in context-dependent strategy use and affective consequences of regulating positive versus negative emotions.

Nevertheless, people do engage in emotion regulation to achieve a variety of goals, not all of which are hedonic in nature (Tamir, 2016). For example, social motives—which are more prevalent in social contexts (Kalokerinos et al., 2017)—may drive the use of social sharing to foster stronger connections with others (Rimé et al., 2020), or the use of suppression to avoid conflict with others (English et al., 2017). Consequently, our finding that high levels of social support were associated with increased sharing and decreased suppression may be partially driven by these socially-oriented goals. Such context-dependent social emotion regulation may be associated with more positive relational outcomes, such as enhanced feelings of closeness, even if it does not predict greater hedonic wellbeing. We see two priorities for future research: first, to examine how various social (and non-social) goals may shape the use and consequences of social emotion-regulation strategies, and second, to determine whether these consequences would be similar across emotions caused by (vs. unrelated to) the interaction partner.

Finally, given the correlational nature of our data, we cannot rule out that our findings regarding context-dependent use of suppression and sharing reflect the opposite causal direction to what we proposed, such that sharing increases social support and suppression decreases social support. Importantly, however, these two interpretations need not be mutually exclusive, but instead may represent a bidirectional loop. Future experimental studies are needed to clarify the directionality of these associations.

### Conclusion

The present research examined how social context in the form of social support shapes the use and short-term affective consequences of two common social emotion-regulation strategies across two experience-sampling studies. Across both studies, we found that higher social support was associated with greater sharing and reduced suppression. However, we did not find robust evidence for context-dependent affective consequences of these regulation strategies. Future large-scale research is warranted to better understand the circumstances in which context-dependent emotion regulation benefits wellbeing, ideally taking into account personal, situational and cultural factors (Doré et al., 2016; Greenaway et al., 2018).

## Supplementary information

ESM 1 (DOCX 132 kb)

## Footnotes

1 SEMA2 is now obsolete and has been replaced by an updated version of the platform, called SEMA3 available at https://sema3.com/

2 We focus exclusively on social sharing and expressive suppression as these were the only fundamentally “social” regulation strategies, which we predicted to vary as a function of social support. The other regulation strategies assessed were: (i) situation selection; (ii) situation modification; (iii) distraction; (iv) rumination; (v) ignoring; (vi) two forms of cognitive reappraisal; and (vii) acceptance.

## Additional Information

### Data and/or Code Availability

The studies and analysis plans were not preregistered. Data and code are publicly available on OSF (https://osf.io/735eg/).
