# Supplementary material for: Social Support Predicts Differential Use, but not Differential Effectiveness, of Expressive Suppression and Social Sharing in Daily Life
Source: Affect Sci. 2022 Aug 22;3(3):641–52. doi: 10.1007/s42761-022-00123-8 (PMC9537407; doi:10.1007/s42761-022-00123-8)
Supplement: Supplementary file 1 — (DOCX 132 kb) [file 42761_2022_123_MOESM1_ESM.docx]

**Supplemental Materials**

**Supplement 1: Overview Measures Included in Study 1**

Below, we report all constructs that were additionally measured in Study 1, as part of a larger research project.

Experience Sampling:

- Social interaction (person they spent most time with)
- Emotion regulation strategies (expressive suppression, social sharing, distraction, situation selection, situation modification, reappraisal, acceptance, rumination, avoidance)
- Context appraisals (control, importance, coping self-efficacy)
- Emotion regulation goals (happy, relaxed, confident, sad, stressed, angry)

Traits:

- Demographics
- Social Isolation
- Depression, Anxiety and Stress
- Perceived Stress
- Self-Esteem
- Personality
- Fear of Negative Evaluation
- Actual and Ideal Affect
- Emotion Regulation
- Self-Control
- Loneliness
- Self-Efficacy
- Self-Compassion
- Implicit Theories and Expectations of Emotion
- Satisfaction with Life
- Interpersonal Support
- Social Desirability

**Supplement 2: Additional Analyses**

**Supplement 2.1: ER Specificity**

**Controlling for the Other ER Strategy (Studies 1 and 2).** To establish whether the effects we observed for suppression and sharing were independent of the other strategy, we repeated our main analyses controlling for the effect of the other regulation strategy in each model. That is, in each model including suppression as an outcome (H1) or a focal predictor (H2 and H3), we added social sharing as a Level-1 covariate, whereas in models including sharing as an outcome (H1) or a focal predictor (H2 and H3) we controlled for the effect of suppression at Level-1 (see Tables S6-S8 for Study 1 and Tables S24-26 for Study 2).

In Study 1, the findings remain largely the same. More specifically, findings for Hypothesis 1 and 2 remain identical. The only difference observed was for the analyses testing Hypothesis 3. As can be seen in Table S8, the interaction effect between Suppression and Social Support predicting NA was no longer significant. Instead, a significant interaction emerged between Social Sharing and Support, indicating that high support buffered the negative effect of sharing on NA.

In Study 2, some small differences emerged. When testing Hypothesis 1 while controlling for the other ER strategy, social support remained a significant predictor of increased sharing, but the negative effect of social support on suppression disappeared. In the analyses testing Hypothesis 2, suppression emerged as a significant negative predictor of valence, in line with our hypothesis. Social sharing remained a non-significant predictor of valence. Finally, no significant interaction effects emerged, replicating our main analyses.

**Non-Social ER Strategies (Studies 1 and 2).** Furthermore, to examine the specificity of our main findings to social – rather than less social – ER strategies, we ran additional analyses examining whether social support predicted differential use of three other ‘non-social’ ER strategies (i.e., reappraisal, rumination, distraction; see Table S9 and S27). We focused on the ER strategies that were measured in both studies, to allow for more robust comparisons. These analyses showed that social support was consistently associated with greater use of reappraisal, but not with rumination (no association in either study) or distraction (positive association in Study 1, but no association in Study 2). Thus, our consistent findings indicating that social support predicts greater sharing and reduced suppression (across both studies) appear to be somewhat specific to these social regulation strategies. Although reappraisal is not generally considered a social emotion-regulation strategy, we speculate that this finding may reflect supportive interaction partners providing alternative interpretations of the emotionally upsetting situation, thus facilitating reappraisal (see Liu et al., 2021; Nils & Rimé, 2012; Sahi et al., 2021).

**Supplement 2.2: Probing Causality: Concurrent, Reversed and Lagged Analyses**

We ran an additional set of analyses to test the temporal nature and directionality of our findings regarding the influence of social support on suppression and sharing. Our main analyses for H1 modelled social support as a predictor of suppression or sharing measured contemporaneously, controlling for lagged suppression or sharing. Given that both social support and sharing/suppression were measured “since the last survey”, we included lagged suppression or sharing as covariates to rule out the possibility that potential associations between social support and these two regulation strategies were, in fact, due to the strategies causing changes in social support.

However, this approach comes with two shortcomings. First, it measures *change* in regulation-strategy use as a function of social support, while we are interested in ER use as a function of the contemporaneous social context, which may be independent of ER use in the previous context. Therefore, we reran our main analyses for Hypothesis 1, examining the use of suppression and sharing as a function of concurrent social support, *without* including lagged ER use as a predictor. These analyses yielded identical findings compared to our main analyses (see Table S10 and S28). Together, our findings show that greater social support in one particular context is associated with less suppression and more social sharing in that same context.
 Second, given that the predictor and outcome variables in our main analyses testing H1 were measured concurrently, we cannot be sure that our findings reflect a causal effect of variation in social support predicting differential use of each regulation strategy, as hypothesized. To test the robustness of this interpretation, we ran additional “lagged” models probing the directionality of the associations of social support, on one hand, and suppression and sharing, on the other.
 To this end, we first ran lagged analyses to examine whether social support at one occasion (T1) would also predict the use of social sharing and suppression reported at the following measurement occasion (T2). In Study 1, we found that social support at T1 positively predicted social sharing at T2, but not suppression at T2 (see Table S11). In Study 2, we found no significant lagged effects for both emotion regulation strategies (see Table S29).

Furthermore, we ran reversed-lagged models, examining whether suppression and social sharing at T1 would predict changes in social support at T2. In both studies, suppression at T1 did not predict social support at T2 (see Table S12 and S30). In Study 1, social sharing at T1 positively predicted social support at T2 (see Table S12), but this effect was not replicated in Study 2 (see Table S30).

Together, these findings suggest that the effect of social support on the use of suppression and sharing may unfold on a relatively short timescale: Our original analyses show that when perceiving high levels of support, participants engaged in more social sharing and less expressive suppression (Table S1 and S21). However, lagged analyses indicated that higher social support did not consistently predict future use of sharing and suppression across both studies. Moreover, although we did find some evidence that higher social support predicted future increases in sharing (in Study 1), there appeared to be a reciprocal effect in the opposite direction, such that greater sharing also predicted future increases in social support. Thus, although we have interpreted our main findings as supporting H1, we cannot rule out the possibility of bidirectional associations among social support, on one hand, and expressive suppression and social sharing, on the other.

**Supplement 2.3: Robustness Checks**

**Valence as an Outcome (Study 1).** To allow comparability between Study 1 and 2, we re-ran our main analyses of Study 1 with valence as the outcome, instead of positive and negative affect separately. Valence was calculated by subtracting negative affect scores from positive affect scores. Supporting Hypothesis 2, these findings show that Suppression was associated with a decrease in valence, whereas Social Sharing was associated with an increase in valence (see Table S13). Furthermore, a significant interaction emerged between suppression and valence, indicating that the negative effect of suppression on valence was buffered for those who perceived high (compared to low) social support (see Table S14). Finally, there was no significant interaction between social sharing and social support predicting valence (see Table S14). Thus, examining our hypotheses with a composite score of emotion (valence), rather than positive and negative affect separately, replicates our main findings of Study 1.

**Negative Emotional Intensity as an Outcome (Study 2).** In addition to our main analyses with bipolar valence (i.e., current affect) as the outcome, we re-ran our analyses using negative emotional intensity ratings of participants’ most negative emotional experience since the last beep. Contrary to our main findings predicting valence, both expressive suppression and social sharing predicted significant increases in negative emotional intensity, meaning that the use of both regulatory strategies was associated with greater negative affect (see Table S31). It should be noted that participants rated the intensity of their most negative emotional experience in the past hour, after which they reported on how they regulated their emotions in response to this emotional experience. These findings may thus indicate that higher negative emotional intensity is an antecedent, rather than consequence, of greater suppression and sharing. In line with our main findings predicting valence, social support did not moderate the effect of either of the two emotion regulation strategies on negative emotional intensity (see Table S32).

**Controlling for Emotional Intensity (Study 2).** Given that people may be more likely to seek (and receive) social support when they experience more intense negative emotions, we reran our analyses of Study 2, controlling for negative emotional intensity of the stressor. When including emotional intensity as covariate in our models testing Hypothesis 1, Social Support was no longer significantly negatively associated with Suppression. In contrast, Social Support remained a significant positive predictor of Social Sharing when controlling for emotional intensity (see Table S33). Including emotional intensity as a covariate in the model did not change our analyses testing Hypothesis 3: Social Support (still) did not moderate the relationship between Suppression and Valence, nor between Social Sharing and Valence (see Table S34).

**Perceived Regulation Success as an Outcome (Study 2).** Given that our findings did not lend strong support for context-dependent affective consequences, we exploratorily examined whether such context-dependent benefits may be primarily reflected in perceptions of effective emotion regulation rather than in actual affective consequences. To this end, we re-ran our analyses testing Hypothesis 3, replacing affect with perceived regulation success as a dependent measure. As can be seen in Table S35, suppression and social sharing did not predict perceived regulation success. We did find a small but significant interaction effect between social support and suppression, but not social sharing. These findings suggest that while people did not experience social sharing as more effective in the context of higher social support (contrary to Hypothesis 3b), they did experience expressive suppression as more effective in the context of *lower* social support. Finally, replicating Study 1 and 2, and in line with a wealth of literature speaking to the importance of perceived responsiveness (see Reis et al., 2017; Reis & Gable, 2015), social support emerged as a significant positive predictor of perceived emotion regulation success.

**Excluding Cases When Alone.** To examine the robustness of our findings, we reran our analyses of both studies excluding cases in which participants reported to be alone. In Study 1, participants answered with whom they spent most of their time since the last survey (“Alone”; “Friends”, “Family”, “Romantic Partner”, “Co-Workers/Classmates”, “Housemate (not family or friends)” or “People you don't know”). We re-ran our analyses of Study 1 excluding the instances in which people indicated having mostly been alone. These analyses yielded identical findings as the findings reported in the manuscript, with one exception (i.e., the interaction between suppression and social support predicting NA is no longer significant). All other findings replicate our main findings, indicating that social support was associated with increased Social Sharing, and decreased Expressive Suppression (see Table S15), but did not moderate the relationship between either of the two emotion regulation strategies and (positive and negative) affect (see Tables S16 and S17).

In our main analyses of Study 2, social support was operationalized such that support was coded as zero when participants indicated to have received no support *and* when they reported being alone. We repeated our analyses excluding “I was alone” responses, such that social support reflected the support participants experienced to have received when in the presence of others. Replicating our main analyses and in line with Hypothesis 1, social support was associated with increased Social Sharing, and decreased Expressive Suppression (see Table S36). Further replicating our main analyses, though inconsistent with Hypothesis 3, social support did not moderate the relationship between either of the two emotion regulation strategies and valence (see Table S37).

**Supplement 2.4: Moderation by Ethnicity**

We explored the potential moderating role of ethnicity, comparing White vs. Asian / South Asian / Mixed / Other. Overall, we found no consistent moderation effects for ethnicity across the two studies. The association between Social Support and Social Sharing did not vary by Ethnicity in Study 1 (see Table S18). In Study 2, we observed only one interaction effect, indicating that social support predicted a stronger increase in social sharing for White participants than for Asian participants (see Table S38) – an effect that we thus did not replicate in Study 1. Similarly, we only observed one interaction effect between Social Support and Ethnicity predicting Suppression in Study 1, indicating that social support was associated with reduced use of suppression among White participants, whereas it was associated with increased use of suppression among South Asian participants (see Table S18). However, we did not replicate this interaction effect in Study 2, where the relationship between Social Support and Suppression was not moderated by any of the ethnicities (see Table S38). Overall, the context-dependent use of social sharing and suppression was thus relatively consistent across participants of various ethnicities.

Ethnicity did not significantly moderate the association between Social Sharing and Affect in Study 1 (see Table S20), with one exception: While Social Sharing was unrelated to NA among White participants, it was associated with reduced NA among those with a mixed ethnicity (see Table S20). We replicated this effect for Valence in Study 2 (see Table S39). Furthermore, Ethnicity significantly interacted with Suppression in predicting Positive and Negative Affect in Study 1, indicating that the negative association between Suppression and PA was stronger among White participants than among South Asian, Asian and Mixed participants, while the positive association between Suppression and NA was stronger among White participants than among South Asian and Mixed participants (see Table S19). However, we did not replicate any of these effects for Valence in Study 2 (see Table S39). In sum, the affective consequences of suppression (more so than social sharing) varied somewhat across ethnicities, though these moderation effects were not consistently observed across both studies.

**Supplement 3: References**

Liu, D. Y., Strube, M. J., & Thompson, R. J. (2021). Interpersonal emotion regulation: An experience sampling study. *Affective Science*, *2*(3), 273–288. https://doi.org/10.1007/s42761-021-00044-y

Nils, F., & Rimé, B. (2012). Beyond the myth of venting: Social sharing modes determine the benefits of emotional disclosure: Effects of sharing modes in emotional disclosure. *European Journal of Social Psychology*, *42*(6), 672–681. https://doi.org/10.1002/ejsp.1880

Reis, H. T., & Gable, S. L. (2015). Responsiveness. *Current Opinion in Psychology*, *1*, 67–71. https://doi.org/10.1016/j.copsyc.2015.01.001

Reis, H. T., Lemay, E. P., & Finkenauer, C. (2017). Toward understanding understanding: The importance of feeling understood in relationships. *Social and Personality Psychology Compass*, *11*(3), 1–22. https://doi.org/10.1111/spc3.12308

Sahi, R. S., Ninova, E., & Silvers, J. A. (2021). With a little help from my friends: Selective social potentiation of emotion regulation. *Journal of Experimental Psychology: General*, *150*(6), 1237–1249. https://doi.org/10.1037/xge0000853

**Supplement 4: Supplemental Tables**

Please find below all supplemental tables as referred to in the main paper or in the Supplemental Materials above.

**Table Overview**

Study 1

- S1: Parameter Estimates for Hypothesis 1a and 1b
- S2: Parameter Estimates for Hypothesis 2a
- S3: Parameter Estimates for Hypothesis 2b
- S4: Parameter Estimates for Hypothesis 3a
- S5: Parameter Estimates for Hypothesis 3b
- S6: Parameter Estimates for Hypothesis 1 while controlling for the other ER strategy (i.e., Predicting Suppression from Social Support, Controlling for Sharing and Predicting Sharing from Social Support, Controlling for Suppression)
- S7: Parameter Estimates for Hypothesis 2 with Expressive Suppression and Social Sharing as Simultaneous Predictors of PA and NA
- S8: Parameter Estimates for Hypothesis 3 with Expressive Suppression and Social Sharing as Simultaneous Predictors of PA and NA
- S9: Parameter Estimates for Hypothesis 1 with Non-Social ER strategies as the Dependent Variable
- S10: Parameter Estimates for Hypothesis 1 without Lagged ER Use as a Predictor
- S11: Parameter Estimates Lagged Analyses Hypothesis 1
- S12: Parameter Estimates Reversed Lagged Analyses Hypothesis 1
- S13: Parameter Estimates for Hypothesis 2 with Valence as the Dependent Variable
- S14: Parameter Estimates for Hypothesis 3 with Valence as the Dependent Variable
- S15: Parameter Estimates for Hypothesis 1 While Excluding Cases When Participants Were Alone
- S16: Parameter Estimates for Hypothesis 3a While Excluding Cases When Participants Were Alone – Study 1
- S17: Parameter Estimates for Hypothesis 3b While Excluding Cases When Participants Were Alone – Study 1
- S18: Parameter Estimates for Hypothesis 1 with Ethnicity as a Moderator – Study 1
- S19: Parameter Estimates for Hypothesis 2a with Ethnicity as a Moderator – Study 1
- S20: Parameter Estimates for Hypothesis 2b with Ethnicity as a Moderator– Study 1

Study 2

- S21: Parameter Estimates Hypothesis 1
- S22: Parameter Estimates Hypothesis 2
- S23: Parameter Estimates Hypothesis 3
- S24: Parameter Estimates for Hypothesis 1 while controlling for the other ER strategy (i.e., Predicting Suppression from Social Support, Controlling for Sharing and Predicting Sharing from Social Support, Controlling for Suppression)
- S25: Parameter Estimates for Hypothesis 2 with Expressive Suppression and Social Sharing as Simultaneous Predictors
- S26: Parameter Estimates for Hypothesis 3 with Expressive Suppression and Social Sharing as Simultaneous Predictors
- S27: Parameter Estimates for Hypothesis 1 with Non-Social ER strategies as the Dependent Variable
- S28: Parameter Estimates for Hypothesis 1 without Lagged ER Use as a Predictor
- S29: Parameter Estimates Lagged Analyses Hypothesis 1
- S30: Parameter Estimates Reversed Lagged Analyses Hypothesis 1
- S31: Parameter Estimates for Hypothesis 2a and 2b with Negative Emotional Intensity with as the Dependent Variable
- S32: Parameter Estimates for Hypothesis 3a and 3b with Negative Emotional Intensity with as the Dependent Variable
- S33: Parameter Estimates for Hypothesis 1a and 1b controlling for Negative Emotional Intensity
- S34: Parameter Estimates for Hypothesis 3a and 3b controlling for Negative Emotional Intensity
- S35: Parameter Estimates for Hypothesis 3 with Perceived Regulation Success as the Dependent Variable
- S36: Parameter Estimates for Hypothesis 1 with Alternative Operationalization of Social Support (i.e., Excluding Cases when Participants were Alone)
- S37: Parameter Estimates for Hypothesis 3 with Alternative Operationalization of Social Support (i.e., Excluding Cases when Participants were Alone)
- S38: Parameter Estimates for Hypothesis 1 with Ethnicity as a Moderator – Study 2
- S39: Parameter Estimates for Hypothesis 2 with Ethnicity as a Moderator – Study 2

Table S1. Parameter Estimates for Hypothesis 1a and 1b – Study 1

|  | **Expressive Suppression** | | | **Social Sharing** | | |
| --- | --- | --- | --- | --- | --- | --- |
| *Predictors* | *Estimate (SE)* | *95% CI* | *p* | *Estimate (SE)* | *95% CI* | *p* |
| Intercept | 38.56 (1.71) | 35.21 – 41.92 | **<0.001** | 35.28 (1.49) | 32.37 – 38.19 | **<0.001** |
| Lagged Suppression | 4.45 (0.38) | 3.71 – 5.19 | **<0.001** |  |  |  |
| Social Support | -1.48 (0.42) | -2.30 – -0.65 | **0.001** | 8.27 (0.45) | 7.38 – 9.16 | **<0.001** |
| Lagged Sharing |  |  |  | 4.34 (0.31) | 3.74 – 4.95 | **<0.001** |
| N | 179 _SEMA_ID_ | | | 178 _SEMA_ID_ | | |
| Observations | 22514 | | | 22345 | | |

Table S2. Parameter Estimates for Hypothesis 2a – Study 1

|  | **Negative Affect** | | | **Positive Affect** | | |
| --- | --- | --- | --- | --- | --- | --- |
| *Predictors* | *Estimate (SE)* | *95% CI* | *p* | *Estimate (SE)* | *95% CI* | *p* |
| Intercept | 22.11 (1.00) | 20.14 – 24.08 | **<0.001** | 63.03 (1.00) | 61.08 – 64.99 | **<0.001** |
| Lagged Negative Affect | 4.84 (0.26) | 4.32 – 5.35 | **<0.001** |  |  |  |
| Suppression | 1.28 (0.17) | 0.96 – 1.61 | **<0.001** | -0.81 (0.19) | -1.19 – -0.43 | **<0.001** |
| Lagged Positive Affect |  |  |  | 5.79 (0.29) | 5.23 – 6.36 | **<0.001** |
| N | 179 _SEMA_ID_ | | | 179 _SEMA_ID_ | | |
| Observations | 22838 | | | 22855 | | |

Table S3. Parameter Estimates for Hypothesis 2b – Study 1

|  | **Negative Affect** | | | **Positive Affect** | | |
| --- | --- | --- | --- | --- | --- | --- |
| *Predictors* | *Estimate (SE)* | *95% CI* | *p* | *Estimate (SE)* | *95% CI* | *p* |
| Intercept | 22.19 (1.00) | 20.23 – 24.16 | **<0.001** | 63.04 (1.00) | 61.08 – 65.00 | **<0.001** |
| Lagged Negative Affect | 5.01 (0.27) | 4.49 – 5.53 | **<0.001** |  |  |  |
| Sharing | -0.05 (0.16) | -0.37 – 0.26 | 0.733 | 1.26 (0.18) | 0.92 – 1.61 | **<0.001** |
| Lagged Positive Affect |  |  |  | 5.84 (0.29) | 5.27 – 6.42 | **<0.001** |
| N | 178 _SEMA_ID_ | | | 178 _SEMA_ID_ | | |
| Observations | 22671 | | | 22691 | | |

Table S4. Parameter Estimates for Hypothesis 3a – Study 1

|  | **Negative Affect** | | | **Positive Affect** | | |
| --- | --- | --- | --- | --- | --- | --- |
| *Predictors* | *Estimate (SE)* | *95% CI* | *p* | *Estimate (SE)* | *95% CI* | *p* |
| Intercept | 22.05 (1.01) | 20.08 – 24.02 | **<0.001** | 63.07 (0.99) | 61.12 – 65.02 | **<0.001** |
| Lagged Negative Affect | 4.56 (0.25) | 4.06 – 5.06 | **<0.001** |  |  |  |
| Suppression | 1.08 (0.14) | 0.80 – 1.36 | **<0.001** | -0.65 (0.16) | -0.96 – -0.33 | **<0.001** |
| Social Support | -2.05 (0.18) | -2.41 – -1.69 | **<0.001** | 3.24 (0.19) | 2.87 – 3.61 | **<0.001** |
| Suppression*Social Support | -0.24 (0.11) | -0.45 – -0.02 | **0.032** | 0.17 (0.11) | -0.06 – 0.39 | 0.142 |
| Lagged Positive Affect |  |  |  | 5.30 (0.27) | 4.77 – 5.82 | **<0.001** |
| N | 179 _SEMA_ID_ | | | 179 _SEMA_ID_ | | |
| Observations | 22664 | | | 22678 | | |

Table S5. Parameter Estimates for Hypothesis 3b – Study 1

|  | **Negative Affect** | | | **Positive Affect** | | |
| --- | --- | --- | --- | --- | --- | --- |
| *Predictors* | *Estimate (SE)* | *95% CI* | *p* | *Estimate (SE)* | *95% CI* | *p* |
| Intercept | 22.23 (1.00) | 20.26 – 24.19 | **<0.001** | 62.96 (1.00) | 61.00 – 64.92 | **<0.001** |
| Lagged Negative Affect | 4.66 (0.25) | 4.17 – 5.16 | **<0.001** |  |  |  |
| Sharing | 0.85 (0.14) | 0.58 – 1.13 | **<0.001** | 0.09 (0.16) | -0.22 – 0.40 | 0.584 |
| Social Support | -2.35 (0.20) | -2.74 – -1.96 | **<0.001** | 3.33 (0.20) | 2.93 – 3.72 | **<0.001** |
| Sharing*Social Support | -0.19 (0.12) | -0.43 – 0.05 | 0.132 | 0.22 (0.13) | -0.04 – 0.47 | 0.100 |
| Lagged Positive Affect |  |  |  | 5.36 (0.27) | 4.83 – 5.89 | **<0.001** |
| N | 178 _SEMA_ID_ | | | 178 _SEMA_ID_ | | |
| Observations | 22501 | | | 22518 | | |

Table S6. Parameter Estimates for Hypothesis 1 while controlling for the other ER strategy (i.e., Social Support predicting Expressive Suppression while controlling for Social Sharing, and Social Support predicting Social Sharing while controlling for Expressive Suppression) – Study 1

|  | **Suppression** | | | **Sharing** | | |
| --- | --- | --- | --- | --- | --- | --- |
| *Predictors* | *Estimate (SE)* | *95% CI* | *p* | *Estimate (SE)* | *95% CI* | *p* |
| Intercept | 38.76 (1.71) | 35.42 – 42.11 | **<0.001** | 35.29 (1.48) | 32.38 – 38.20 | **<0.001** |
| Lagged Suppression | 4.19 (0.36) | 3.48 – 4.89 | **<0.001** |  |  |  |
| Sharing | -0.91 (0.43) | -1.76 – -0.06 | **0.038** |  |  |  |
| Social Support | -1.17 (0.34) | -1.83 – -0.52 | **0.001** | 7.73 (0.43) | 6.89 – 8.58 | **<0.001** |
| Lagged Sharing |  |  |  | 4.07 (0.30) | 3.49 – 4.65 | **<0.001** |
| Suppression |  |  |  | -0.74 (0.42) | -1.55 – 0.08 | 0.079 |
| N | 178 _SEMA_ID_ | | | 178 _SEMA_ID_ | | |
| Observations | 22308 | | | 22304 | | |

Table S7. Parameter Estimates for Hypothesis 2 with Expressive Suppression and Social Sharing as Simultaneous Predictors of PA and NA – Study 1

|  | **Negative Affect** | | | **Positive Affect** | | |
| --- | --- | --- | --- | --- | --- | --- |
| *Predictors* | *Estimate (SE)* | *95% CI* | *p* | *Estimate (SE)* | *95% CI* | *p* |
| Intercept | 22.20 (1.00) | 20.24 – 24.17 | **<0.001** | 63.03 (1.00) | 61.07 – 64.99 | **<0.001** |
| Lagged Negative Affect | 4.83 (0.26) | 4.31 – 5.34 | **<0.001** |  |  |  |
| Sharing | -0.02 (0.15) | -0.31 – 0.27 | 0.887 | 1.11 (0.17) | 0.78 – 1.43 | **<0.001** |
| Suppression | 1.32 (0.15) | 1.02 – 1.62 | **<0.001** | -0.92 (0.17) | -1.26 – -0.58 | **<0.001** |
| Lagged Positive Affect |  |  |  | 5.68 (0.29) | 5.12 – 6.25 | **<0.001** |
| N | 178 _SEMA_ID_ | | | 178 _SEMA_ID_ | | |
| Observations | 22585 | | | 22601 | | |

Table S8. Parameter Estimates for Hypothesis 3 with Expressive Suppression, Social Sharing and Social Support as Simultaneous Predictors of PA and NA – Study 1

|  | **Negative Affect** | | | **Positive Affect** | | |
| --- | --- | --- | --- | --- | --- | --- |
| *Predictors* | *Estimate (SE)* | *95% CI* | *p* | *Estimate (SE)* | *95% CI* | *p* |
| Intercept | 22.21 (1.00) | 20.25 – 24.18 | **<0.001** | 62.97 (1.00) | 61.01 – 64.93 | **<0.001** |
| Lagged Negative Affect | 4.52 (0.25) | 4.03 – 5.01 | **<0.001** |  |  |  |
| Suppression | 0.82 (0.14) | 0.54 – 1.10 | **<0.001** | 0.04 (0.16) | -0.26 – 0.35 | 0.796 |
| Sharing | -2.28 (0.19) | -2.65 – -1.91 | **<0.001** | 3.26 (0.20) | 2.88 – 3.64 | **<0.001** |
| Social Support | 1.14 (0.14) | 0.87 – 1.40 | **<0.001** | -0.72 (0.16) | -1.03 – -0.42 | **<0.001** |
| Suppression*Social Support | -0.15 (0.12) | -0.39 – 0.08 | 0.199 | 0.18 (0.13) | -0.07 – 0.44 | 0.161 |
| Sharing*Social Support | -0.24 (0.12) | -0.46 – -0.01 | **0.044** | 0.18 (0.11) | -0.05 – 0.40 | 0.124 |
| Lagged Positive Affect |  |  |  | 5.24 (0.27) | 4.72 – 5.77 | **<0.001** |
| N | 178 _SEMA_ID_ | | | 178 _SEMA_ID_ | | |
| Observations | 22458 | | | 22472 | | |

Table S9: Parameter Estimates for Hypothesis 1 with Non-Social ER strategies as the Dependent Variable

|  | **Rumination** | | | | | **Reappraisal** | | | | | **Distraction** | | | |
| --- | --- | --- | --- | --- | --- | --- | --- | --- | --- | --- | --- | --- | --- | --- |
| *Predictors* | *Estimate (SE)* | *95% CI* | *p* | | *Estimate (SE)* | | *95% CI* | | *p* | *Estimate (SE)* | | *95% CI* | *p* | |
| Intercept | 36.62 (1.65) | 33.39 – 39.85 | **<0.001** | | 40.17 (1.64) | | 36.95 – 43.39 | | **<0.001** | 51.93 (1.65) | | 48.71 – 55.16 | **<0.001** | |
| Lagged Rumination | 4.87 (0.36) | 4.17 – 5.57 | **<0.001** | |  | |  | |  |  | |  |  | |
| Social Support | 0.61 (0.33) | -0.05 – 1.26 | 0.070 | | 3.03 (0.27) | | 2.49 – 3.56 | | **<0.001** | 1.11 (0.34) | | 0.43 – 1.78 | **0.002** | |
| Lagged Reappraisal |  |  |  | | 3.78 (0.26) | | 3.28 – 4.29 | | **<0.001** |  | |  |  | |
| Lagged Distraction |  |  | |  | |  |  |  | | 4.88 (0.35) | | 4.18 – 5.57 | **<0.001** | |
| N | 179 _SEMA_ID_ | | | | | 179 _SEMA_ID_ | | | | | 179 _SEMA_ID_ | | |  |
| Observations | 22499 | | | | | 22398 | | | | | 22507 | | | |

Table S10. Parameter Estimates for Hypothesis 1 without Lagged ER Use as a Predictor – Study 1

|  | **Suppression** | | | **Sharing** | | |
| --- | --- | --- | --- | --- | --- | --- |
| *Predictors* | *Estimate (SE)* | *95% CI* | *p* | *Estimate (SE)* | *95% CI* | *p* |
| Intercept | 38.52 (1.70) | 35.19 – 41.85 | **<0.001** | 35.35 (1.48) | 32.45 – 38.25 | **<0.001** |
| Social Support | -1.55 (0.45) | -2.43 – -0.68 | **0.001** | 8.97 (0.47) | 8.05 – 9.89 | **<0.001** |
| N | 179 _SEMA_ID_ | | | 179 _SEMA_ID_ | | |
| Observations | 28868 | | | 28872 | | |

Table S11. Parameter Estimates for Lagged Analyses Hypothesis 1 – Study 1

|  | **Suppression** | | | **Sharing** | | |
| --- | --- | --- | --- | --- | --- | --- |
| *Predictors* | *Estimate (SE)* | *95% CI* | *p* | *Estimate (SE)* | *95% CI* | *p* |
| Intercept | 38.59 (1.71) | 35.24 – 41.95 | **<0.001** | 35.21 (1.49) | 32.30 – 38.13 | **<0.001** |
| Lagged Suppression | 4.69 (0.40) | 3.92 – 5.47 | **<0.001** |  |  |  |
| Lagged Social Support | -0.26 (0.22) | -0.70 – 0.17 | 0.233 | 1.66 (0.22) | 1.23 – 2.08 | **<0.001** |
| Lagged Sharing |  |  |  | 5.21 (0.35) | 4.53 – 5.88 | **<0.001** |
| N | 179 _SEMA_ID_ | | | 178 _SEMA_ID_ | | |
| Observations | 22502 | | | 22335 | | |

Table S12. Parameter Estimates Reversed Lagged Analyses Hypothesis 1 – Study 1

|  | **Social Support** | | | **Social Support** | | |
| --- | --- | --- | --- | --- | --- | --- |
| *Predictors* | *Estimate (SE)* | *95% CI* | *p* | *Estimate (SE)* | *95% CI* | *p* |
| Intercept | 56.08 (1.23) | 53.68 – 58.49 | **<0.001** | 56.03 (1.23) | 53.62 – 58.43 | **<0.001** |
| Lagged Social Support | 6.65 (0.35) | 5.96 – 7.34 | **<0.001** | 6.25 (0.35) | 5.55 – 6.95 | **<0.001** |
| Lagged Suppression | -0.11 (0.22) | -0.54 – 0.31 | 0.597 |  |  |  |
| Lagged Sharing |  |  |  | 1.26 (0.23) | 0.81 – 1.70 | **<0.001** |
| N | 179 _SEMA_ID_ | | | 178 _SEMA_ID_ | | |
| Observations | 22402 | | | 22243 | | |

Table S13: Parameter Estimates for Hypothesis 2 with Valence as the Dependent Variable

|  | **Valence** | | | **Valence** | | |
| --- | --- | --- | --- | --- | --- | --- |
| *Predictors* | *Estimate (SE)* | *95% CI* | *p* | *Estimate (SE)* | *95% CI* | *p* |
| Intercept | 40.95 (1.71) | 37.59 – 44.30 | **<0.001** | 40.87 (1.72) | 37.50 – 44.24 | **<0.001** |
| Lagged Valence | 10.35 (0.53) | 9.31 – 11.40 | **<0.001** | 10.62 (0.54) | 9.56 – 11.68 | **<0.001** |
| Suppression | -2.04 (0.33) | -2.68 – -1.40 | **<0.001** |  |  |  |
| Sharing |  |  |  | 1.30 (0.30) | 0.71 – 1.90 | **<0.001** |
| N | 179 _SEMA_ID_ | | | 178 _SEMA_ID_ | | |
| Observations | 22805 | | | 22638 | | |

Table S14: Parameter Estimates for Hypothesis 3 with Valence as the Dependent Variable

|  | **Valence** | | | **Valence** | | |
| --- | --- | --- | --- | --- | --- | --- |
| *Predictors* | *Estimate (SE)* | *95% CI* | *p* | *Estimate (SE)* | *95% CI* | *p* |
| Intercept | 41.04 (1.71) | 37.68 – 44.39 | **<0.001** | 40.74 (1.72) | 37.37 – 44.11 | **<0.001** |
| Lagged Valence | 9.57 (0.50) | 8.58 – 10.56 | **<0.001** | 9.77 (0.51) | 8.77 – 10.76 | **<0.001** |
| Suppression | -1.69 (0.27) | -2.22 – -1.16 | **<0.001** |  |  |  |
| Social Support | 5.22 (0.34) | 4.55 – 5.89 | **<0.001** | 5.59 (0.37) | 4.87 – 6.31 | **<0.001** |
| Suppression*Social Support | 0.42 (0.21) | 0.02 – 0.82 | **0.043** |  |  |  |
| Sharing |  |  |  | -0.77 (0.27) | -1.29 – -0.25 | **0.004** |
| Sharing*Social Support |  |  |  | 0.43 (0.23) | -0.03 – 0.88 | 0.072 |
| N | 179 _SEMA_ID_ | | | 178 _SEMA_ID_ | | |
| Observations | 22631 | | | 22469 | | |

Table S15: Parameter Estimates for Hypothesis 1 While Excluding Cases When Participants Were Alone – Study 1

|  | **Suppression** | | | **Sharing** | | |
| --- | --- | --- | --- | --- | --- | --- |
| *Predictors* | *Estimate (SE)* | *95% CI* | *p* | *Estimate (SE)* | *95% CI* | *p* |
| Intercept | 39.44 (1.64) | 36.23 – 42.66 | **<0.001** | 38.13 (1.50) | 35.18 – 41.08 | **<0.001** |
| Lagged Suppression | 3.87 (0.39) | 3.10 – 4.65 | **<0.001** |  |  |  |
| Social Support | -2.93 (0.46) | -3.83 – -2.03 | **<0.001** | 7.79 (0.49) | 6.82 – 8.75 | **<0.001** |
| Lagged Sharing |  |  |  | 4.28 (0.35) | 3.60 – 4.96 | **<0.001** |
| N | 179 _SEMA_ID_ | | | 178 _SEMA_ID_ | | |
| Observations | 14577 | | | 14501 | | |

S16: Parameter Estimates for Hypothesis 3a While Excluding Cases When Participants Were Alone – Study 1

|  | **Negative Affect** | | | **Positive Affect** | | |
| --- | --- | --- | --- | --- | --- | --- |
| *Predictors* | *Estimate (SE)* | *95% CI* | *p* | *Estimate (SE)* | *95% CI* | *p* |
| Intercept | 22.17 (0.99) | 20.23 – 24.10 | **<0.001** | 62.88 (0.99) | 60.93 – 64.83 | **<0.001** |
| Lagged Negative Affect | 4.29 (0.26) | 3.77 – 4.81 | **<0.001** |  |  |  |
| Suppression | 0.97 (0.16) | 0.65 – 1.29 | **<0.001** | -0.59 (0.18) | -0.94 – -0.23 | **0.001** |
| Social Support | -2.69 (0.24) | -3.16 – -2.21 | **<0.001** | 3.95 (0.26) | 3.45 – 4.46 | **<0.001** |
| Suppression*Social Support | -0.12 (0.14) | -0.40 – 0.15 | 0.380 | 0.10 (0.14) | -0.18 – 0.38 | 0.494 |
| Lagged Positive Affect |  |  |  | 5.04 (0.27) | 4.52 – 5.56 | **<0.001** |
| N | 179 _SEMA_ID_ | | | 179 _SEMA_ID_ | | |
| Observations | 14678 | | | 14696 | | |

S17: Parameter Estimates for Hypothesis 3b While Excluding Cases When Participants Were Alone – Study 1

|  | **Negative Affect** | | | **Positive Affect** | | |
| --- | --- | --- | --- | --- | --- | --- |
| *Predictors* | *Estimate (SE)* | *95% CI* | *p* | *Estimate (SE)* | *95% CI* | *p* |
| Intercept | 22.29 (0.98) | 20.37 – 24.22 | **<0.001** | 62.74 (1.00) | 60.79 – 64.69 | **<0.001** |
| Lagged Negative Affect | 4.38 (0.27) | 3.86 – 4.90 | **<0.001** |  |  |  |
| Sharing | 0.74 (0.15) | 0.44 – 1.05 | **<0.001** | 0.10 (0.17) | -0.24 – 0.44 | 0.567 |
| Social Support | -3.02 (0.25) | -3.52 – -2.53 | **<0.001** | 4.09 (0.26) | 3.57 – 4.61 | **<0.001** |
| Sharing*Social Support | -0.03 (0.15) | -0.32 – 0.27 | 0.850 | 0.02 (0.15) | -0.28 – 0.31 | 0.908 |
| Lagged Positive Affect |  |  |  | 5.10 (0.27) | 4.58 – 5.63 | **<0.001** |
| N | 178 _SEMA_ID_ | | | 178 _SEMA_ID_ | | |
| Observations | 14596 | | | 14618 | | |

S18: Parameter Estimates for Hypothesis 1 with Ethnicity as a Moderator – Study 1

|  | **Suppression** | | | **Sharing** | | |
| --- | --- | --- | --- | --- | --- | --- |
| *Predictors* | *Estimate (SE)* | *95% CI* | *p* | *Estimate (SE)* | *95% CI* | *p* |
| Intercept | 30.49 (2.13) | 26.31 – 34.68 | **<0.001** | 29.02 (1.93) | 25.23 – 32.81 | **<0.001** |
| Lagged Suppression | 4.45 (0.38) | 3.71 – 5.19 | **<0.001** |  |  |  |
| Social Support | -2.50 (0.56) | -3.60 – -1.39 | **<0.001** | 8.10 (0.62) | 6.88 – 9.32 | **<0.001** |
| South Asian (vs. White) | 27.39 (6.12) | 15.40 – 39.39 | **<0.001** | 18.19 (5.54) | 7.33 – 29.06 | **0.001** |
| Other (vs. White) | 11.47 (6.59) | -1.44 – 24.38 | 0.083 | 8.50 (5.97) | -3.19 – 20.20 | 0.156 |
| Mixed (vs. White) | -3.14 (8.10) | -19.01 – 12.74 | 0.699 | 4.60 (7.34) | -9.78 – 18.98 | 0.532 |
| Asian (vs. White) | 18.56 (3.55) | 11.61 – 25.51 | **<0.001** | 14.19 (3.22) | 7.89 – 20.49 | **<0.001** |
| Social Support*South Asian | 3.74 (1.61) | 0.58 – 6.90 | **0.021** | -0.08 (1.78) | -3.57 – 3.41 | 0.964 |
| Social Support*Other | 2.41 (1.73) | -0.98 – 5.80 | 0.165 | 0.66 (1.91) | -3.08 – 4.40 | 0.729 |
| Social Support*Mixed | 3.04 (2.14) | -1.15 – 7.22 | 0.157 | 4.62 (2.37) | -0.02 – 9.26 | 0.053 |
| Social Support*Asian | 1.61 (0.93) | -0.21 – 3.44 | 0.085 | -0.14 (1.03) | -2.16 – 1.88 | 0.893 |
| Lagged Sharing |  |  |  | 4.33 (0.31) | 3.73 – 4.94 | **<0.001** |
| N | 179 _SEMA_ID_ | | | 178 _SEMA_ID_ | | |
| Observations | 22514 | | | 22345 | | |

S19: Parameter Estimates for Hypothesis 2a with Ethnicity as a Moderator – Study 1

|  | **Negative Affect** | | | **Positive Affect** | | |
| --- | --- | --- | --- | --- | --- | --- |
| *Predictors* | *Estimate (SE)* | *95% CI* | *p* | *Estimate (SE)* | *95% CI* | *p* |
| Intercept | 20.72 (1.35) | 18.08 – 23.36 | **<0.001** | 62.59 (1.35) | 59.95 – 65.23 | **<0.001** |
| Lagged Negative Affect | 4.83 (0.26) | 4.32 – 5.35 | **<0.001** |  |  |  |
| Suppression | 1.59 (0.23) | 1.15 – 2.04 | **<0.001** | -1.41 (0.25) | -1.91 – -0.92 | **<0.001** |
| South Asian (vs. White) | 2.53 (3.84) | -4.99 – 10.05 | 0.511 | 8.99 (3.88) | 1.38 – 16.60 | **0.022** |
| Other (vs. White) | 6.17 (4.13) | -1.93 – 14.27 | 0.137 | -5.23 (4.18) | -13.42 – 2.96 | 0.213 |
| Mixed (vs. White) | -6.91 (5.08) | -16.87 – 3.05 | 0.176 | 6.14 (5.14) | -3.94 – 16.21 | 0.234 |
| Asian (vs. White) | 3.71 (2.22) | -0.65 – 8.07 | 0.097 | -0.43 (2.25) | -4.84 – 3.98 | 0.849 |
| Suppression*South Asian | -1.68 (0.64) | -2.94 – -0.42 | **0.010** | 3.19 (0.72) | 1.79 – 4.59 | **<0.001** |
| Suppression*Other | 0.17 (0.69) | -1.17 – 1.52 | 0.800 | 0.40 (0.77) | -1.10 – 1.91 | 0.601 |
| Suppression*Mixed | -2.32 (0.85) | -3.99 – -0.65 | **0.007** | 2.28 (0.95) | 0.43 – 4.14 | **0.017** |
| Suppression*Asian | -0.34 (0.37) | -1.07 – 0.39 | 0.359 | 0.85 (0.42) | 0.03 – 1.66 | **0.043** |
| Lagged Positive Affect |  |  |  | 5.79 (0.29) | 5.22 – 6.35 | **<0.001** |
| N | 179 _SEMA_ID_ | | | 179 _SEMA_ID_ | | |
| Observations | 22838 | | | 22855 | | |

S20: Parameter Estimates for Hypothesis 2b with Ethnicity as a Moderator – Study 1

|  | **Negative Affect** | | | **Positive Affect** | | |
| --- | --- | --- | --- | --- | --- | --- |
| *Predictors* | *Estimate (SE)* | *95% CI* | *p* | *Estimate (SE)* | *95% CI* | *p* |
| Intercept | 20.86 (1.35) | 18.21 – 23.51 | **<0.001** | 62.56 (1.36) | 59.90 – 65.22 | **<0.001** |
| Lagged Negative Affect | 5.01 (0.27) | 4.49 – 5.53 | **<0.001** |  |  |  |
| Sharing | 0.21 (0.22) | -0.22 – 0.63 | 0.344 | 0.92 (0.24) | 0.44 – 1.39 | **<0.001** |
| South Asian (vs. White) | 2.38 (3.85) | -5.16 – 9.92 | 0.537 | 9.14 (3.89) | 1.51 – 16.76 | **0.020** |
| Other (vs. White) | 6.01 (4.14) | -2.10 – 14.13 | 0.148 | -5.09 (4.19) | -13.30 – 3.12 | 0.226 |
| Mixed (vs. White) | -7.18 (5.09) | -17.16 – 2.80 | 0.160 | 6.30 (5.15) | -3.80 – 16.40 | 0.223 |
| Asian (vs. White) | 3.61 (2.23) | -0.77 – 7.98 | 0.108 | -0.40 (2.26) | -4.83 – 4.03 | 0.859 |
| Sharing*South Asian | 0.39 (0.61) | -0.82 – 1.59 | 0.530 | 0.60 (0.69) | -0.75 – 1.95 | 0.382 |
| Sharing*Other | -0.64 (0.66) | -1.94 – 0.65 | 0.329 | 0.66 (0.74) | -0.78 – 2.10 | 0.372 |
| Sharing*Mixed | -2.39 (0.82) | -4.00 – -0.78 | **0.004** | 1.69 (0.92) | -0.11 – 3.49 | 0.067 |
| Sharing*Asian | -0.51 (0.36) | -1.21 – 0.19 | 0.155 | 0.63 (0.40) | -0.15 – 1.41 | 0.113 |
| Lagged Positive Affect |  |  |  | 5.84 (0.29) | 5.27 – 6.42 | **<0.001** |
| N | 178 _SEMA_ID_ | | | 178 _SEMA_ID_ | | |
| Observations | 22671 | | | 22691 | | |

Table S21: Parameter Estimates for Hypothesis 1a and 1b – Study 2

|  | **Suppression** | | | **Sharing** | | |
| --- | --- | --- | --- | --- | --- | --- |
| *Predictors* | *Estimate (SE)* | *95% CI* | *p* | *Estimate (SE)* | *95% CI* | *p* |
| Intercept | 37.24 (1.74) | 33.83 – 40.64 | **<0.001** | 41.45 (1.66) | 38.20 – 44.70 | **<0.001** |
| Lagged Suppression | 2.97 (0.47) | 2.05 – 3.89 | **<0.001** |  |  |  |
| Social Support | -1.13 (0.47) | -2.05 – -0.21 | **0.017** | 3.97 (0.50) | 3.00 – 4.94 | **<0.001** |
| Lagged Sharing |  |  |  | 2.66 (0.49) | 1.70 – 3.61 | **<0.001** |
| N | 120 _sema_id_ | | | 120 _sema_id_ | | |
| Observations | 4272 | | | 4264 | | |

Table S22: Parameter Estimates for Hypothesis 2a and 2b – Study 2

|  | **Valence** | | | **Valence** | | |
| --- | --- | --- | --- | --- | --- | --- |
| *Predictors* | *Estimate (SE)* | *95% CI* | *p* | *Estimate (SE)* | *95% CI* | *p* |
| Intercept | 3.05 (0.27) | 2.52 – 3.57 | **<0.001** | 3.05 (0.27) | 2.53 – 3.57 | **<0.001** |
| Lagged Valence | 0.82 (0.09) | 0.66 – 0.99 | **<0.001** | 0.81 (0.09) | 0.64 – 0.99 | **<0.001** |
| Suppression | -0.16 (0.09) | -0.34 – 0.02 | 0.086 |  |  |  |
| Sharing |  |  |  | -0.01 (0.10) | -0.19 – 0.18 | 0.941 |
| N | 122 _sema_id_ | | | 122 _sema_id_ | | |
| Observations | 4421 | | | 4416 | | |

Table S23: Parameter Estimates for Hypothesis 3a and 3b – Study 2

|  | | **Valence** | | | | **Valence** | | | | |
| --- | --- | --- | --- | --- | --- | --- | --- | --- | --- | --- |
| *Predictors* | *Estimate (SE)* | | *95% CI* | | *p* | | *Estimate (SE)* | *95% CI* | *p* | |
| Intercept | 3.03 (0.27) | | 2.49 – 3.56 | | **<0.001** | | 3.03 (0.27) | 2.50 – 3.56 | **<0.001** | |
| Lagged Valence | 0.78 (0.08) | | 0.61 – 0.94 | | **<0.001** | | 0.77 (0.09) | 0.60 – 0.94 | **<0.001** | |
| Suppression | -0.13 (0.09) | | -0.31 – 0.05 | | 0.151 | |  |  |  | |
| Social Support | 0.62 (0.07) | | 0.48 – 0.77 | | **<0.001** | | 0.66 (0.08) | 0.50 – 0.83 | **<0.001** | |
| Suppression* Social Support | -0.01 (0.06) | | -0.13 – 0.11 | | 0.905 | |  |  |  | |
| Sharing |  | |  | |  | -0.15 (0.10) | | -0.34 – 0.04 | 0.118 | |
| Sharing*Social Support |  | | |  |  | 0.03 (0.07) | | -0.11 – 0.16 | 0.683 | |
| N | 120 _sema_id_ | | | | | 120 _sema_id_ | | | | |
| Observations | 4334 | | | | | 4331 | | | |  |

Table S24: Parameter Estimates for Hypothesis 1 while controlling for the other ER strategy (i.e., Social Support predicting Expressive Suppression while controlling for Social Sharing, and Social Support predicting Social Sharing while controlling for Expressive Suppression) – Study 2

|  | **Suppression** | | | **Sharing** | | | |
| --- | --- | --- | --- | --- | --- | --- | --- |
| *Predictors* | *Estimate (SE)* | *95% CI* | *p* | | *Estimate (SE)* | *95% CI* | *p* |
| Intercept | 37.27 (1.74) | 33.87 – 40.68 | **<0.001** | | 41.55 (1.66) | 38.30 – 44.79 | **<0.001** |
| Lagged Suppression | 2.46 (0.46) | 1.56 – 3.36 | **<0.001** | |  |  |  |
| Sharing | -2.52 (0.79) | -4.07 – -0.98 | **0.002** | |  |  |  |
| Social Support | -0.57 (0.44) | -1.44 – 0.29 | 0.198 | | 3.63 (0.47) | 2.71 – 4.54 | **<0.001** |
| Lagged Sharing |  |  |  | | 2.19 (0.49) | 1.24 – 3.14 | **<0.001** |
| Suppression |  |  |  | | -2.60 (0.79) | -4.14 – -1.06 | **0.001** |
| N | 120 _sema_id_ | | | | 120 _sema_id_ | | |
| Observations | 4261 | | | | 4257 | | |

Table S25. Parameter Estimates for Hypothesis 2 with Expressive Suppression and Social Sharing as Simultaneous Predictors of Valence – Study 2

|  | **Valence** | | |
| --- | --- | --- | --- |
| *Predictors* | *Estimate (SE)* | *95% CI* | *p* |
| Intercept | 3.07 (0.27) | 2.55 – 3.59 | **<0.001** |
| Lagged Valence | 0.79 (0.08) | 0.63 – 0.95 | **<0.001** |
| Sharing | -0.02 (0.09) | -0.21 – 0.16 | 0.795 |
| Suppression | -0.18 (0.09) | -0.35 – -0.01 | **0.041** |
| N _sema_id_ | 122 | | |
| Observations | 4405 | | |

Table S26. Parameter Estimates for Hypothesis 3 with Expressive Suppression, Social Sharing and Social Support as Simultaneous Predictors of Valence – Study 2

|  | **Valence** | | |
| --- | --- | --- | --- |
| *Predictors* | *Estimate (SE)* | *95% CI* | *p* |
| Intercept | 3.04 (0.27) | 2.50 – 3.57 | **<0.001** |
| Lagged Valence | 0.83 (0.06) | 0.72 – 0.94 | **<0.001** |
| Sharing | -0.18 (0.06) | -0.29 – -0.06 | **0.003** |
| Social Support | 0.65 (0.06) | 0.53 – 0.77 | **<0.001** |
| Suppression | -0.17 (0.06) | -0.28 – -0.06 | **0.003** |
| Sharing*Social Support | 0.01 (0.05) | -0.09 – 0.12 | 0.778 |
| Suppression*Social Support | 0.01 (0.05) | -0.09 – 0.11 | 0.864 |
| N _sema_id_ | 120 | | |
| Observations | 4320 | | |

*Note.* Due to convergence issues, this model only included fixed slopes.

Table S27: Parameter Estimates for Hypothesis 1 with Non-Social ER strategies as the Dependent Variable

|  | **Rumination** | | | | **Reappraisal** | | | | **Distraction** | | | | | |
| --- | --- | --- | --- | --- | --- | --- | --- | --- | --- | --- | --- | --- | --- | --- |
| *Predictors* | | *Estimate (SE)* | *95% CI* | *p* | *Estimate (SE)* | *95% CI* | | *p* | | *Estimate (SE)* | | *95% CI* | | *p* |
| Intercept | | 40.02 (1.73) | 36.62 – 43.42 | **<0.001** | 42.35 (1.86) | 38.70 – 46.00 | | **<0.001** | | 50.26 (1.70) | | 46.94 – 53.58 | | **<0.001** |
| Lagged Rumination | | 3.19 (0.43) | 2.36 – 4.03 | **<0.001** |  |  | |  | |  | |  | |  |
| Social Support | | -0.67 (0.47) | -1.59  – 0.26 | 0.161 | 1.38 (0.42) | 0.55 – 2.21 | | **0.002** | | -0.52 (0.53) | | -1.56  – 0.52 | | 0.327 |
| Lagged Reappraisal | |  |  |  | 2.59 (0.37) | 1.87 – 3.32 | | **<0.001** | |  |  | | |  |
| Lagged Distraction | |  |  |  |  |  |  | | | 3.71 (0.48) | | 2.77 – 4.66 | **<0.001** | |
| N | 120 _sema_id_ | | | | 120 _sema_id_ | | | | 120 _sema_id_ | | | | | |
| Observations | 4263 | | | | 4260 | | | | 4253 | | | | | |

Table S28. Parameter Estimates for Hypothesis 1 without Lagged ER Use as a Predictor – Study 2

|  | **Suppression** | | | **Sharing** | | |
| --- | --- | --- | --- | --- | --- | --- |
| *Predictors* | *Estimate (SE)* | *95% CI* | *p* | *Estimate (SE)* | *95% CI* | *p* |
| Intercept | 37.20 (1.69) | 33.89 – 40.50 | **<0.001** | 41.66 (1.62) | 38.48 – 44.84 | **<0.001** |
| Social Support | -0.90 (0.40) | -1.69 – -0.11 | **0.028** | 4.15 (0.46) | 3.25 – 5.04 | **<0.001** |
| N | 120 _sema_id_ | | | 120 _sema_id_ | | |
| Observations | 5767 | | | 5766 | | |

Table S29. Parameter Estimates for Lagged Analyses Hypothesis 1 – Study 2

|  | **Suppression** | | | **Sharing** | | |
| --- | --- | --- | --- | --- | --- | --- |
| *Predictors* | *Estimate (SE)* | *95% CI* | *p* | *Estimate (SE)* | *95% CI* | *p* |
| Intercept | 37.18 (1.72) | 33.80 – 40.56 | **<0.001** | 41.36 (1.66) | 38.11 – 44.61 | **<0.001** |
| Lagged Suppression | 3.06 (0.48) | 2.13 – 4.00 | **<0.001** |  |  |  |
| Lagged Social Support | 0.21 (0.35) | -0.47 – 0.89 | 0.541 | 0.31 (0.37) | -0.41 – 1.03 | 0.399 |
| Lagged Sharing |  |  |  | 2.97 (0.51) | 1.98 – 3.96 | **<0.001** |
| N | 120 _sema_id_ | | | 120 _sema_id_ | | |
| Observations | 4271 | | | 4264 | | |

Table S30. Parameter Estimates for Reverse Lagged Analyses Hypothesis 1 – Study 2

|  | **Social Support** | | | **Social Support** | | |
| --- | --- | --- | --- | --- | --- | --- |
| *Predictors* | *Estimate (SE)* | *95% CI* | *p* | *Estimate (SE)* | *95% CI* | *p* |
| Intercept | 19.94 (1.57) | 16.86 – 23.03 | **<0.001** | 20.08 (1.57) | 17.00 – 23.16 | **<0.001** |
| Lagged Social Support | 4.25 (0.67) | 2.94 – 5.56 | **<0.001** | 4.16 (0.68) | 2.83 – 5.49 | **<0.001** |
| Lagged Suppression | 0.16 (0.48) | -0.77 – 1.09 | 0.738 |  |  |  |
| Lagged Sharing |  |  |  | 0.48 (0.46) | -0.43 – 1.39 | 0.301 |
| N | 120 _sema_id_ | | | 120 _sema_id_ | | |
| Observations | 4311 | | | 4311 | | |

Table S31: Parameter Estimates for Hypothesis 2a and 2b with Negative Emotional Intensity with as the Dependent Variable – Study 2

|  | **Negative Emotion** | | | **Negative Emotion** | | |
| --- | --- | --- | --- | --- | --- | --- |
| *Predictors* | *Estimate (SE)* | *95% CI* | *p* | *Estimate (SE)* | *95% CI* | *p* |
| Intercept | 39.29 (1.33) | 36.68 – 41.90 | **<0.001** | 39.26 (1.33) | 36.66 – 41.86 | **<0.001** |
| Lagged Negative Emotion | 4.35 (0.51) | 3.35 – 5.35 | **<0.001** | 4.19 (0.49) | 3.23 – 5.15 | **<0.001** |
| Suppression | 3.75 (0.55) | 2.66 – 4.83 | **<0.001** |  |  |  |
| Sharing |  |  |  | 2.94 (0.61) | 1.74 – 4.13 | **<0.001** |
| N | 122 _sema_id_ | | | 122 _sema_id_ | | |
| Observations | 4401 | | | 4395 | | |

Table S32: Parameter Estimates for Hypothesis 3a and 3b with Negative Emotional Intensity with as the Dependent Variable – Study 2

|  | **Negative Emotion** | | | **Negative Emotion** | | |
| --- | --- | --- | --- | --- | --- | --- |
| *Predictors* | *Estimate (SE)* | *95% CI* | *p* | *Estimate (SE)* | *95% CI* | *p* |
| Intercept | 39.13 (1.35) | 36.48 – 41.77 | **<0.001** | 39.15 (1.34) | 36.51 – 41.78 | **<0.001** |
| Lagged Negative Emotion | 4.15 (0.50) | 3.17 – 5.13 | **<0.001** | 3.93 (0.46) | 3.02 – 4.84 | **<0.001** |
| Suppression | 3.54 (0.55) | 2.46 – 4.63 | **<0.001** |  |  |  |
| Social Support | -2.19 (0.50) | -3.17 – -1.21 | **<0.001** | -2.98 (0.54) | -4.05 – -1.91 | **<0.001** |
| Support*Social Support | -0.06 (0.35) | -0.75 – 0.63 | 0.858 |  |  |  |
| Sharing |  |  |  | 3.62 (0.62) | 2.41 – 4.83 | **<0.001** |
| Sharing*Social Support |  |  |  | -0.37 (0.42) | -1.20 – 0.45 | 0.377 |
| N | 120 _sema_id_ | | | 120 _sema_id_ | | |
| Observations | 4315 | | | 4311 | | |

Table S33: Parameter Estimates for Hypothesis 1a and 1b controlling for Negative Emotional Intensity

|  | **Expressive Suppression** | | | **Sharing** | | |
| --- | --- | --- | --- | --- | --- | --- |
| *Predictors* | *Estimate (SE)* | *95% CI* | *p* | *Estimate (SE)* | *95% CI* | *p* |
| Intercept | 37.35 (1.74) | 33.94 – 40.76 | **<0.001** | 41.51 (1.65) | 38.27 – 44.75 | **<0.001** |
| Lagged Suppression | 2.66 (0.47) | 1.75 – 3.58 | **<0.001** |  |  |  |
| Emotional Intensity | 3.18 (0.51) | 2.17 – 4.18 | **<0.001** | 3.23 (0.56) | 2.14 – 4.32 | **<0.001** |
| Social Support | -0.69 (0.44) | -1.56 – 0.17 | 0.120 | 4.27 (0.50) | 3.28 – 5.26 | **<0.001** |
| Lagged Sharing |  |  |  | 2.30 (0.43) | 1.46 – 3.14 | **<0.001** |
| N | 120 _sema_id_ | | | 120 _sema_id_ | | |
| Observations | 4272 | | | 4264 | | |

Table S34: Parameter Estimates for Hypothesis 3a and 3b controlling for Negative Emotional Intensity

|  | **Valence** | | | **Valence** | | |
| --- | --- | --- | --- | --- | --- | --- |
| *Predictors* | *Estimate (SE)* | *95% CI* | *p* | *Estimate (SE)* | *95% CI* | *p* |
| Intercept | 3.00 (0.27) | 2.47 – 3.53 | **<0.001** | 2.99 (0.27) | 2.46 – 3.52 | **<0.001** |
| Lagged Valence | 0.54 (0.07) | 0.41 – 0.68 | **<0.001** | 0.56 (0.08) | 0.41 – 0.71 | **<0.001** |
| Emotional Intensity | -1.42 (0.11) | -1.64 – -1.19 | **<0.001** | -1.39 (0.11) | -1.61 – -1.17 | **<0.001** |
| Suppression | 0.09 (0.07) | -0.05 – 0.23 | 0.210 |  |  |  |
| Social Support | 0.45 (0.06) | 0.33 – 0.58 | **<0.001** | 0.43 (0.07) | 0.30 – 0.57 | **<0.001** |
| Suppression*Social Support | -0.02 (0.05) | -0.11 – 0.08 | 0.733 |  |  |  |
| Sharing |  |  |  | 0.07 (0.07) | -0.06 – 0.20 | 0.290 |
| Sharing*Social Support |  |  |  | -0.01 (0.05) | -0.10 – 0.09 | 0.888 |
| N | 120 _sema_id_ | | | 120 _sema_id_ | | |
| Observations | 4334 | | | 4331 | | |

Table S35. Parameter Estimates for Hypothesis 3 with Perceived Regulation Success as the Dependent Variable – Study 2

|  | **Perceived Regulation Success** | | | **Perceived Regulation Success** | | |
| --- | --- | --- | --- | --- | --- | --- |
| *Predictors* | *Estimate (SE)* | *95% CI* | *p* | *Estimate (SE)* | *95% CI* | *p* |
| Intercept | 65.58 (1.34) | 62.94 – 68.21 | **<0.001** | 65.50 (1.33) | 62.89 – 68.10 | **<0.001** |
| Lagged Success | 3.49 (0.46) | 2.58 – 4.40 | **<0.001** | 3.33 (0.42) | 2.51 – 4.16 | **<0.001** |
| Suppression | 0.06 (0.51) | -0.93 – 1.06 | 0.902 |  |  |  |
| Social Support | 2.62 (0.38) | 1.89 – 3.36 | **<0.001** | 2.82 (0.41) | 2.02 – 3.63 | **<0.001** |
| Suppression*Social Support | -0.63 (0.31) | -1.25 – -0.02 | **0.048** |  |  |  |
| Sharing |  |  |  | -0.46 (0.62) | -1.67 – 0.76 | 0.463 |
| Sharing*Social Support |  |  |  | 0.21 (0.33) | -0.43 – 0.85 | 0.516 |
| N | 120 _sema_id_ | | | 120 _sema_id_ | | |
| Observations | 4249 | | | 4250 | | |

Table S36: Parameter Estimates for Hypothesis 1a and 1b with Alternative Operationalization of Social Support (i.e., Excluding Cases when Participants were Alone) – Study 2

|  | **Expressive Suppression** | | | **Sharing** | | |
| --- | --- | --- | --- | --- | --- | --- |
| *Predictors* | *Estimate (SE)* | *95% CI* | *p* | *Estimate (SE)* | *95% CI* | *p* |
| Intercept | 39.13 (1.72) | 35.76 – 42.50 | **<0.001** | 44.77 (1.67) | 41.50 – 48.04 | **<0.001** |
| Lagged Suppression | 3.09 (0.63) | 1.85 – 4.33 | **<0.001** |  |  |  |
| Social Support | -3.14 (0.63) | -4.38 – -1.90 | **<0.001** | 4.13 (0.63) | 2.89 – 5.36 | **<0.001** |
| Lagged Sharing |  |  |  | 2.38 (0.69) | 1.03 – 3.74 | **0.001** |
| N | 120 _sema_id_ | | | 120 _sema_id_ | | |
| Observations | 2264 | | | 2261 | | |

Table S37: Parameter Estimates for Hypothesis 3a and 3b with Alternative Operationalization of Social Support (i.e., Excluding Cases when Participants were Alone) – Study 2

|  | **Valence** | | | **Valence** | | |
| --- | --- | --- | --- | --- | --- | --- |
| *Predictors* | *Estimate (SE)* | *95% CI* | *p* | *Estimate (SE)* | *95% CI* | *p* |
| Intercept | 3.21 (0.28) | 2.67 – 3.75 | **<0.001** | 3.24 (0.27) | 2.71 – 3.78 | **<0.001** |
| Lagged Valence | 0.67 (0.11) | 0.46 – 0.88 | **<0.001** | 0.67 (0.11) | 0.45 – 0.89 | **<0.001** |
| Suppression | -0.08 (0.10) | -0.28 – 0.11 | 0.404 |  |  |  |
| Social Support | 0.96 (0.10) | 0.77 – 1.16 | **<0.001** | 1.04 (0.12) | 0.82 – 1.27 | **<0.001** |
| Suppression*Social Support | -0.04 (0.08) | -0.19 – 0.12 | 0.630 |  |  |  |
| Sharing |  |  |  | -0.27 (0.12) | -0.50 – -0.03 | **0.028** |
| Sharing*Social Support |  |  |  | 0.11 (0.10) | -0.09 – 0.31 | 0.297 |
| N | 120 _sema_id_ | | | 120 _sema_id_ | | |
| Observations | 2294 | | | 2295 | | |

S38: Parameter Estimates for Hypothesis 1 with Ethnicity as a Moderator – Study 2

|  | **Expressive Suppression** | | | **Social Sharing** | | |
| --- | --- | --- | --- | --- | --- | --- |
| *Predictors* | *Estimate (SE)* | *95% CI* | *p* | *Estimate (SE)* | *95% CI* | *p* |
| Intercept | 36.60 (4.13) | 28.51 – 44.70 | **<0.001** | 34.11 (3.93) | 26.40 – 41.82 | **<0.001** |
| Lagged Suppression | 2.97 (0.47) | 2.05 – 3.89 | **<0.001** |  |  |  |
| Social Support | -1.69 (1.16) | -3.97 – 0.59 | 0.150 | 6.31 (1.15) | 4.05 – 8.57 | **<0.001** |
| Asian (vs. White) | 0.44 (4.70) | -8.78 – 9.65 | 0.926 | 8.45 (4.48) | -0.32 – 17.23 | 0.062 |
| Other (vs. White) | -7.94 (9.40) | -26.37 – 10.48 | 0.400 | 5.39 (8.95) | -12.15 – 22.94 | 0.548 |
| South Asian (vs. White) | -0.17 (6.27) | -12.46 – 12.11 | 0.978 | 10.45 (5.97) | -1.25 – 22.15 | 0.083 |
| Mixed (vs. White) | 14.50 (8.79) | -2.72 – 31.72 | 0.102 | 12.92 (8.37) | -3.48 – 29.32 | 0.125 |
| Social Support*Asian | 0.57 (1.32) | -2.02 – 3.15 | 0.668 | -3.62 (1.31) | -6.18 – -1.06 | **0.007** |
| Social Support*Other | 1.49 (2.54) | -3.48 – 6.47 | 0.558 | -2.10 (2.52) | -7.03 – 2.83 | 0.406 |
| Social Support*South Asian | 0.85 (1.70) | -2.47 – 4.17 | 0.618 | -1.88 (1.68) | -5.16 – 1.41 | 0.266 |
| Social Support*Mixed | 0.47 (2.38) | -4.19 – 5.13 | 0.843 | 2.79 (2.36) | -1.84 – 7.42 | 0.241 |
| Lagged Social Sharing |  |  |  | 2.64 (0.49) | 1.69 – 3.60 | **<0.001** |
| N | 120 _sema_id_ | | | 120 _sema_id_ | | |
| Observations | 4272 | | | 4264 | | |

S39: Parameter Estimates for Hypothesis 2 with Ethnicity as a Moderator – Study 2

|  | **Valence** | | | **Valence** | | |
| --- | --- | --- | --- | --- | --- | --- |
| *Predictors* | *Estimate (SE)* | *95% CI* | *p* | *Estimate (SE)* | *95% CI* | *p* |
| Intercept | 3.95 (0.60) | 2.77 – 5.14 | **<0.001** | 3.95 (0.60) | 2.77 – 5.13 | **<0.001** |
| Lagged Valence | 0.82 (0.09) | 0.66 – 0.99 | **<0.001** | 0.81 (0.09) | 0.64 – 0.99 | **<0.001** |
| Suppression | -0.17 (0.22) | -0.61 – 0.26 | 0.432 |  |  |  |
| Asian (vs. White) | -1.70 (0.69) | -3.05 – -0.36 | **0.015** | -1.68 (0.68) | -3.02 – -0.34 | **0.016** |
| Other (vs. White) | 2.08 (1.38) | -0.62 – 4.77 | 0.134 | 2.05 (1.37) | -0.64 – 4.74 | 0.138 |
| South Asian (vs. White) | 0.72 (0.90) | -1.05 – 2.48 | 0.428 | 0.70 (0.90) | -1.06 – 2.46 | 0.436 |
| Mixed (vs. White) | -1.67 (1.29) | -4.19 – 0.86 | 0.198 | -1.65 (1.28) | -4.17 – 0.86 | 0.201 |
| Suppression*Asian | 0.00 (0.25) | -0.49 – 0.49 | 0.993 |  |  |  |
| Suppression*Other | 0.03 (0.49) | -0.94 – 1.00 | 0.949 |  |  |  |
| Suppression*South Asian | 0.07 (0.32) | -0.56 – 0.70 | 0.834 |  |  |  |
| Suppression*Mixed | 0.05 (0.49) | -0.91 – 1.00 | 0.921 |  |  |  |
| Sharing |  |  |  | 0.01 (0.22) | -0.43 – 0.44 | 0.972 |
| Sharing*Asian |  |  |  | 0.06 (0.25) | -0.43 – 0.55 | 0.803 |
| Sharing*Other |  |  |  | -0.24 (0.49) | -1.20 – 0.71 | 0.619 |
| Sharing*South Asian |  |  |  | -0.64 (0.32) | -1.27 – -0.00 | 0.052 |
| Sharing*Mixed |  |  |  | 1.06 (0.46) | 0.15 – 1.96 | **0.024** |
| N | 122 _sema_id_ | | | 122 _sema_id_ | | |
| Observations | 4421 | | | 4416 | | |
